# Supplementary material for: Next-generation biomonitoring of the early-life chemical exposome in neonatal and infant development
Source: Nat Commun. 2022 May 12;13:2653. doi: 10.1038/s41467-022-30204-y (PMC9098442; doi:10.1038/s41467-022-30204-y)
Supplement: Supplementary file 1 — Supplementary Information File [file 41467_2022_30204_MOESM1_ESM.pdf]

Supplementary Information

Next-generation biomonitoring of the early-life chemical exposome in neonatal and infant development

Thomas Jamnik[1], Mira Flasch[1], Dominik Braun[1], Yasmin Fareed[1], Daniel Wasinger[1], David Seki[2,3], David Berry[3], Angelika Berger[2], Lukas Wisgrill[2,4], Benedikt Warth[1,4,*]

[1]University of Vienna, Faculty of Chemistry, Department of Food Chemistry and Toxicology, Währingerstraße 38, 1090 Vienna, Austria
[2]Medical University of Vienna, Division of Neonatology, Pediatric Intensive Care and Neuropediatrics, Comprehensive Center for Pediatrics, Währinger Gürtel 18-20, 1090 Vienna, Austria
[3]Centre for Microbiology and Environmental Systems Science, Department of Microbiology and Ecosystem Science, Division of Microbial Ecology, University of Vienna, 1090 Vienna, Austria
[4]Exposome Austria, Research Infrastructure and National EIRENE Hub, Austria

*Correspondence: benedikt.warth@univie.ac.at

Table of contents

Chemicals and Reagents 2

LC-MS/MS method development 2

Internal standards 2

Comparison of plasma and serum matrices 3

Supplementary Figure 1: Comparison between plasma and serum chromatograms, and the presence of nicotine metabolites in breast milk 4

Supplementary Table 1: Information on suppliers and additional data on chemicals and reagents used in the study. 5

Supplementary Table 2: Analyte-specific LC-MS/MS parameters 8

Supplementary Table 3: Spiking levels and extraction outcomes 11

Supplementary Table 4: Calibration, sensitivity, matrix effects and ion ratios 14

Supplementary Table 5: Validation outcomes for all included chemicals. 17

Supplementary Table 6: Comparison with published multi-class assays 21

Chemicals and Reagents

Supplementary Table 1 provides supplier information and CAS numbers for reagents and chemicals used in this work. Analytical standards were initially dissolved in appropriate solvents (acetonitrile (ACN), methanol, LC-MS-grade water or dimethyl sulfoxide) to yield concentrations of 1 or 2 mg mL^{-1}. A stock solution containing all compounds at 20-fold the concentration of the highest standard was prepared in ACN. Calibration standards were separately prepared for each validation study from this standard mixture. A separate solution containing all internal standards (IS) at 20-fold the fortified concentrations was prepared in ACN. All chemical stocks and solutions were stored at -20°C. The commercial, analytical-grade perfluorooctanesulfonic acid standard consisted of at least 3 isomers with the linear structure eluting last at the same time as the internal standard. Similarly, a racemic mixture of 2 diastereomers resulted in 2 separated peaks for anisodamine. For both compounds, isomeric peaks were integrated together.

LC-MS/MS method development

The xenoestrogen method developed by Preindl et al. (2019)[1] also incorporated endogenous estrogens. While the objective of this method covered general xenobiotic assessment, the decision was made to also include estrogens in the method development as the base for future methods and to report technical parameters to the scientific community. Moreover, parameters and validation results of xenobiotics that were not successfully validated in any matrix are also included.

Precursor ions, tandem MS fragment masses, and optimized compound-dependent ion optic parameters (declustering potential (DP), collision energy (CE), cell exit potential (CXP)) of the mass spectrometer were determined to achieve maximal ion transmission by injecting single compound standards (ranging from 50 µg L^{-1} to 5 mg L^{-1} in 10% ACN) directly into the ion source and utilizing the automatic optimization tool of the system. Final compound-dependent multiple reaction monitoring (MRM) parameters are summarized in Supplementary Table 2. Parameters of the electrospray ionization (ESI) source (curtain gas flow (CUR), sheath gas flow (GS1), drying gas flow (GS2), temperature, spray voltages in positive and negative mode) and the dissociation gas pressure (CAD) were optimized for a selection of the most representative compounds by comparison of ion transmission rates and signal-to-noise ratios (S/N) at different configurations using a flow injection analysis of solvent standards (in ACN) without chromatographic separation as the column was replaced by a connecting piece. Afterwards, full LC-MS/MS measurements of matrix standards were conducted and the S/N ratios of all analytes were evaluated at different configurations. The final method utilized CUR at 30 psi, GS1 at 80 psi, GS2 at 60 psi, a temperature of 500°C, a negative ion spray voltage of -4500 V, a positive ion spray voltage of 5500 V and the CAD pressure setting at "medium". The position of the vertical probe yielded best signal-to-noise ratios at 2 mm instead of the standard setting of 5 mm.

Internal standards

Isotopically-labelled standards were spiked at the following concentrations: IS ethylparaben, IS butylparaben, IS methylparaben, IS propylparaben, IS genistein: 0.5 ng mL^{-1}; IS bisphenol A (BPA), IS estradiol (E2), IS perfluorooctanesulfonic acid (PFOS), IS perfluorooctanoic acid (PFOA), IS zearalenone (ZEN): 1 ng mL^{-1}; IS mono-2-ethylhexyl phthalate (MEHP), IS mono-n-butyl phthalate (MBP): 2 ng mL^{-1}, IS 4-tert-octylphenol: 10 ng mL^{-1}, IS p-hydroxybenzoic acid (pOHBA): 30 ng mL^{-1}. Internal standard calibration was conducted for methyl-, ethyl-, propyl-, butylparaben, BPA, genistein, ZEN, PFOA, PFOS, E2, MBP and pOHBA in all matrices, for 4-tert-OP in serum and breast milk and for MEHP in urine and serum. Internal calibration was not conducted for 4-tert-OP in urine and MEHP in breast milk because of low IS recovery. The applied spiking concentrations were chosen to reliably

detect the IS, but also to avoid wasting needlessly high quantities in order to keep the method cost-effective and therefore suitable for large-scale applications.

Comparison of plasma and serum matrices

The commercially-available heparinized pooled plasma was heavily contaminated with a number of toxicologically-relevant analytes (bisphenol A (BPA), 2-naphthol, perfluorooctanoic acid (PFOA), perfluorooctanesulfonic acid (PFOS), mono-n-ethylhexyl phthalate (MEHP), cotinine, trans-3-hydroxy cotinine) which resulted in a much higher baseline signal when the extracted matrix (matrix blank) was compared to the pooled serum, thus calibration could not encompass low-level contaminations and would have hampered the quantitation of trace amounts. In a few cases, such as PFOS, the highest matrix-matched standard was of similar intensity to the extracted plasma blank. Fortifying at even higher concentrations would have resolved this issue for highly-contaminated samples, however, the purpose of this method was the accurate quantitation of trace-level contamination. For MEHP, the blank contamination even resulted in detector saturation. Matrix contamination was also present in serum, but fewer analytes were affected. Except for cotinine and MEHP, the contaminations were acceptably low to enable sufficient linear calibration and consequently accurate quantitation after applying baseline correction. This highlights the difficulty of appropriate matrix selection to determine ubiquitous environmental xenobiotics that heavily contaminate commercially-available pooled matrices. Moreover, qualitative features (retention time, ion ratios) were similar for both matrices. Consequently, a decision was made to apply a standard calibration with serum as the matrix of choice for method development and quantitation of plasma samples of prematurely-born babies, as this resembled the infant samples more closely than solvent standards and did not exhibit the heavy background contamination observed with the pooled plasma.

Supplementary Figure 1 depicts chromatograms of selected compounds in the blank serum and plasma matrices and also the fortified standards. As a result of the high contamination, contrary to serum, the plasma blank could not be differentiated from the highest matrix-matched standard for perfluorooctanoic acid (Supplementary Figure 1 a). Similarly, matrix contamination of the plasma was notably higher for bisphenol A and 2-naphthol when compared to serum (Supplementary Figure 1 b, c). No quantitation would have been possible for the isomers of PFOS using plasma-matched calibration. Supplementary Figure 1 d-h compares chromatograms of both mass transitions (quantifier and qualifier) of selected compounds that were identified in the premature infant cohort between both matrices.

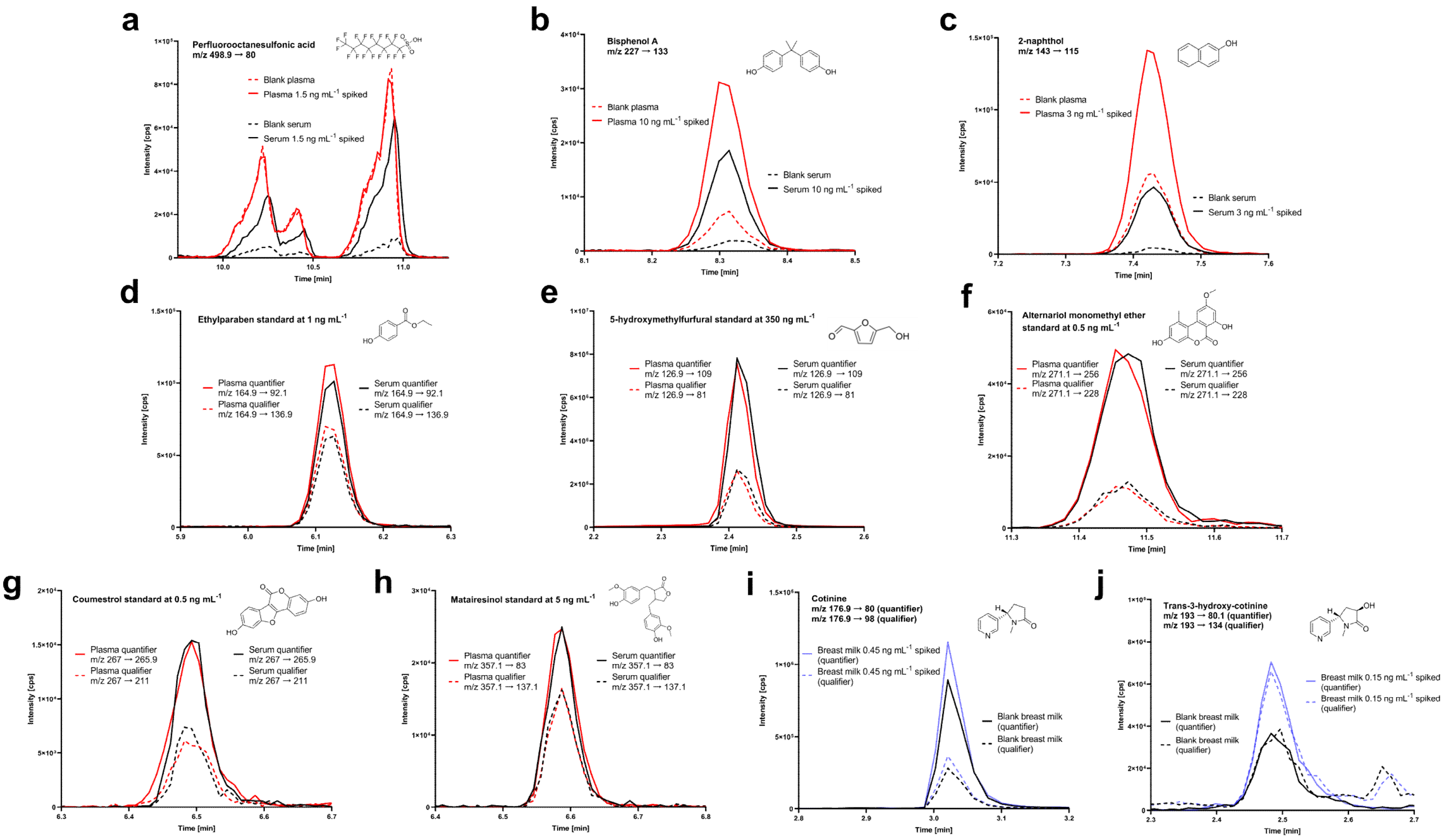

Supplementary Figure 1: Comparison between plasma and serum chromatograms, and the presence of nicotine metabolites in breast milk.
a-c Comparison of non-spiked plasma and serum (plasma/serum blank) and the highest spiked concentration level of the respective matrix calibration series. **d-h** Depiction of both mass transitions of chosen compounds detected and quantitated in the premature infant cohort in a serum and a plasma standard at the same fortified concentration. **i-j** Evidence of the presence of nicotine metabolites in the blank breast milk matrix that was used for method development. Source data are provided as a Source Data file.

Supplementary Table 1: Information on suppliers and additional data on chemicals and reagents used in the study.

Compound	CAS	Catalogue number	Supplier	Lot number
$^{13}C_{12}$-Bisphenol A (BPA)	263261-85-0	720186-5.00mg	Sigma Aldrich	MBBB1923V
$^{13}C_{18}$-Zearalenone (ZEN)			Romer Labs	
$^{13}C_2$-Mono-*n*-butyl phthalate (MBP)		CLM-4590-MT-1.2	Cambridge Isotope Laboratories	SDIC-008
$^{13}C_2$-mono-2-ethylhexyl phthalate (MEHP)		CLM-4584-MT-1.2	Cambridge Isotope Laboratories	SDHC-027
$^{13}C_3$-Estradiol (E2)	1261254-48-1	719552-1mg	Sigma Aldrich	MBBC4575
13C6-4-*tert*-octylphenol (4-*tert*-OP)	1173020-24-0	33565	Sigma Aldrich	BCBW5639
$^{13}C_6$-Butylparaben		32125	Sigma Aldrich	BCBT7363
$^{13}C_6$-Ethylparaben		32125	Sigma Aldrich	BCBT7363
$^{13}C_6$-*p*-hydroxybenzoic acid (*p*OHBA)	267399-29-5	587869-10.0mg	Sigma Aldrich	MBBB9644V
$^{13}C_6$-Propylparaben		32125	Sigma Aldrich	BCBT7363
$^{13}C_8$-Perfluorooctanoic acid (PFOA)		ES-5571	Cambridge Isotope Laboratories	PR-28266
$^{13}C_8$-Perfluorooctanesulfonic acid (PFOS)		ES-5571	Cambridge Isotope Laboratories	PR-28266
16-Epiestriol (16EpiE3)	547-81-9	E586500	TorontoResearchChemicals	29-AZC-158-2
16-α-Hydroxyestrone (16OHE1)	566-76-7	H941900	TorontoResearchChemicals	3 CGF-676
17-Epiestriol (17EpiE3)	1228-72-4	E586510	TorontoResearchChemicals	20-THT-39-2
1-Hydroxypyrene	5315-79-7	H952700	TorontoResearchChemicals	8-BHW-120-2
2-*tert*-Butylphenol (2-*tert*-BP)	88-18-6	B99405-50mL	Sigma Aldrich	SHBD7123V
2-Hydroxyestradiol (2OHE2)	362-05-0	H941890	TorontoResearchChemicals	5-SBT 161-4
2-Methoxyestradiol (2MeOE2)	362-07-2	89242-1mg	Sigma Aldrich	BCBT7859
2-Methoxyestrone (2MeOE1)	362-08-3	73532-25mg	Sigma Aldrich	BCBS2816V
2-Naphthol	135-19-3	185507-5g	Sigma Aldrich	BCBV0179
3-Benzylidencamphor (3-BC)	15087-24-8	91556-25mg	Sigma Aldrich	BCBT9983
3-Hydroxyphenanthrene	605-87-8	P295010	TorontoResearchChemicals	3-GRS-168-1
4-Hydroxyestrone (4OHE1)	3131-23-5	H941950	TorontoResearchChemicals	5KH 128-1
4-Methoxyestradiol (4MeOE2)	26788-23-8	M262630	TorontoResearchChemicals	4-YKZ-7-1
4-Methoxyestrone (MeOE1)	58562-33-7	M226135	TorontoResearchChemicals	1SRE-175-1
4-Methylbenzyliden camphor (4-MBC)	36861-47-9	61547-50mg	Sigma Aldrich	BCBS7570V
4-Octylphenol (4-OP)	1806-26-4	384445-1g	Sigma Aldrich	MKBW6803V
4-*tert*-Octylphenol (4-*tert*-OP)	140-66-9	442858	Sigma Aldrich	LRAB8559
5-Hydroxymethylfurfural (HMF)	67-47-0	W501808	Sigma Aldrich	STBJ7088
5-Hydroxymethyl-2-furanoic acid (HMFA)	6338-41-6	H947020	TorontoResearchChemicals	2-JMO-50-1
8-Prenylnaringenin (8-Pn)	53846-50-7	75119	Sigma Aldrich	BCBR2514V
α-Zearalanol (α-ZAL)	26538-44-3		Sigma Aldrich	
α-Zearalenol (α-ZEL)	36455-72-8	S0242	RomerLabs	L14424Z
α-Zearalenol-14-glucuronide (α-ZEL-GlcA)	Synthesized at the Technical University of Vienna and kindly provided by Dr. Mikula			
Alternariol	641-38-3	A575760	Sigma Aldrich	
Alternariol monomethyl ether (AME)	26894-49-5	A575770	TorontoResearchChemicals	10-CGS-13-1
Acrylamide	79-06-1	A191300	TorontoResearchChemicals	8-ABY-165-1
Anisodamine	55869-99-3	SML0252	Sigma Aldrich	0000049069
Aristolactam I	13395-02-3	A771200	TorontoResearchChemicals	1-AKS-87-1
Aristolochic acid I	313-67-7	A771300	TorontoResearchChemicals	1-JLW-39-1
Benzophenone 1	131-56-6	126217-100G	Sigma Aldrich	BCBN3480V
Benzophenone 2	131-55-5	T16403-25g	Sigma Aldrich	MKBN6515V
Benzyl butyl phthalate	85-68-7	442503	Sigma Aldrich	LC26422V

Compound	CAS	Catalogue number	Supplier	Lot number
Benzylparaben	94-18-8	07389-100mg	Sigma Aldrich	BCBR26688V
β-Zearalanol (β-ZAL)	42422-68-4		Sigma Aldrich	
β-Zearalenol (β-ZEL)	71030-11-0	S0243	RomerLabs	L15241D
β-Zearalenol-14-glucuronide (β-ZEL-GlcA)	Synthesized at the Technical University of Vienna and kindly provided by Dr. Mikula			
Bisphenol A (BPA)	80-05-07	239658	Sigma Aldrich	MKBS0991V
Bisphenol AF (BPAF)	1478-61-1	90477-100mg	Sigma Aldrich	BCBV2156
Bisphenol B (BPB)	77-40-7	50877-100mg	Sigma Aldrich	BCBS0967
Bisphenol C (BPC)	79-97-0	68118-100mg	Sigma Aldrich	BCBV2464
Bisphenol F (BPF)	620-92-8	51453-100mg	Sigma Aldrich	BCBT7018
Bisphenol S (BPS)	80-09-1	43034-100mg	Sigma Aldrich	BCBV2462
Bromoacetic acid	79-08-3	B679075	TorontoResearchChemicals	1-CAL-99-1
Butylparaben	94-26-8	PHR1022-1g	Sigma Aldrich	LRAB3701
Cotinine	486-56-6	74003	Sigma Aldrich	BCCC4550
Coumestrol	479-13-0	BML-S180-0005	Enzo Life sciences	11071708
d4-Genistein	187960-08-3	D-6282	CDN Isotopes	k-457
Daidzein	486-66-8	D7802	Sigma Aldrich	013M4027V
Dibromoacetic acid	631-64-1	442551	Sigma Aldrich	LRAC3539
Dibutyl phthalate	84-74-2	18281-50mg	Sigma Aldrich	BCBV9940
Dichloroacetic acid	79-43-6	36545	Sigma Aldrich	BCBW5201
Estradiol-17-glucuronide (E2-17-GlcA)	15087-02-2	E1127	Sigma Aldrich	4856
Enterodiol	80226-00-2	45198-1MG-F	Sigma Aldrich	BCBT3858
Enterolactone	78473-71-9	45199-1mg-F	Sigma Aldrich	BCBT6193
Equol	94105-90-5	13184	Cayman Chemical Company	0433202-6
Estradiol (E2)	50-28-2	E8875	Sigma Aldrich	SLBP6339V
Estradiol-3-sulfate (E2-3-sulfate)	4999-79-5	E9505-25mg	Sigma Aldrich	067M4032V
Estriol (E3)	50-27-1	E1253	Sigma Aldrich	115H0256
Estrone (E1)	53-16-7	46573-250mg	Sigma Aldrich	SZBE205XV
Ethinylestradiol	57-63-6	E685100	TorontoResearchChemicals	1-TIM-118-1
Ethylparaben	120-47-8	PHR1011-1g	Sigma Aldrich	LRAB3628
Fenarimol	60168-88-9	45484-250mg	Sigma Aldrich	BCBW4607
Formononetin	485-72-3	94334-50mg	Sigma Aldrich	BCBT8620
Genistein	446-72-0	1097	Extrasynthese	Batch24ID: 0421/0
Glycidamide	5694-00-8	G615250	TorontoResearchChemicals	12-YMK-158-4
Glycitein	40957-83-3	G635400	TorontoResearchChemicals	4-GRS-54-1
Isobutylparaben	4247-02-3	715077-25g	Sigma Aldrich	23283
Isoxanthohumol	70872-29-6	1367S	Extrasynthese	Batch01ID: 1020/0
Jacobine	6870-67-3	6219.88	PhytoPlan	18101105
Jacobine-N-oxide	38710-25-7	6222.96	PhytoPlan	19010306
Matairesinol	580-72-3	40043-5MG-F	Sigma Aldrich	BCBW3093
Methiocarb	2032-65-7	36152-100mg	Sigma Aldrich	SZBF106XV
Methylparaben	99-76-3	47889	Sigma Aldrich	LRAB6911
Mono-2-ethylhexyl phthalate (MEHP)	4376-20-9	796832-500mg	Sigma Aldrich	MKCD4270
Mono-*n*-butyl phthalate (MBP)	131-70-4	30751-100mg	Sigma Aldrich	BCBT4836
N-butylbenzenesulfonamide	3622-84-2	B90653-250mL	Sigma Aldrich	MKCD8065
N-nitrosodimethylamine (NDMA)	62-75-9	CRM40059	Sigma Aldrich	LRAC5069
Nonylphenol	84852-15-3	46018-1g	Sigma Aldrich	BCBT5112
Octyl methoxycinnamate (OMC)	5466-77-3	55529-100mg	Sigma Aldrich	BCBQ5953V
Perfluorooctanoic acid (PFOA)	335-67-1	171468-5g	Sigma Aldrich	MKCC6736
Perfluorooctanesulfonic acid (PFOS)	1763-23-1	33607	Sigma Aldrich	BCBW0899

Compound	CAS	Catalogue number	Supplier	Lot number
PhIP	105650-23-5	A617000	TorontoResearchChemicals	7-RCD-119-3
p-Hydroxybenzoic acid (*p*OHBA)	99-96-7	H5376	Sigma Aldrich	090K8911
Prochloraz	67747-09-5	45631-250mg	Sigma Aldrich	BCBW4694
Propylparaben	94-13-3	PHR1010-1G	Sigma Aldrich	LRAB2246
Resveratrol	501-36-0	R5010	Sigma Aldrich	SLBC6832V
Riddelliin	23246-96-0	6312.98	PhytoPlan	17100501
Riddelliin-N-oxide	75056-11-0	6313.97	PhytoPlan	17100601
Scopolamine	51-34-3	S200005	TorontoResearchChemicals	11-DPM-182-2
Tetrabromobisphenol A (TBPA)	79-94-7	11223-100mg	Sigma Aldrich	BCBW5493
Trans-3-hydroxy-cotinine	34834-67-8	H924500	TorontoResearchChemicals	2-WHH-123-9
Triclosan	3380-34-5	93453-100mg	Sigma Aldrich	BCBS1844
Xanthohumol	569-83-5	13496S	Extrasynthese	Batch03ID: 0524/0
Zearalanone (ZAN)	5975-78-0		Sigma Aldrich	
Zearalenone (ZEN)	17924-92-4	001009	RomerLabs	S13114Z
Zearalenone-14-glucuronide (ZEN-14 GlcA)	Synthesized at the Technical University of Vienna and kindly provided by Dr. Mikula			
Zearalenone-14-sulfate (ZEN-14-sulfate)	Gift from Prof. Berthiller from the University of Natural Resources and Life Sciences Vienna			
**Reagent**	**CAS**	**Catalogue number**	**Supplier**	**Lot number**
Ammonium fluoride	12125-01-8	52481-50g	Honeywell Fluka	H0790
LC-MS grade water		83645.320	VWR chemicals	various
LC-MS grade acetonitrile (ACN)	75-05-8	34967	Honeywell Riedel-del Haen	various
LC-MS grade methanol (MeOH)	67-56-1	34966	Honeywell Riedel-del Haen	various
Magnesium sulfate (anhydrous)	7487-88-9	413485000	Acros organics	A0379632
Sodium chloride	7647-14-5	9268.1	Roth	406250348
Heparinized pooled human AB plasma		IPLAWBLIH100ML	Innovative Research	31982
Pooled human AB serum		H4522	Sigma Aldrich	SLBW8068

Supplementary Table 2: Analyte-specific LC-MS/MS parameters for xenobiotics and endogenous estrogens.
Apart from $^{13}C_{12}$-BPA and $^{13}C_{18}$-ZEN, internal standards were not individually tuned and ion optic parameters were adapted from the non-labelled compounds. For some analytes, retention times varied between validation batches and the three matrices urine (U), serum (S) and breast milk (M).

Compound	MS/MS parameters						Retention time [min]
	Polarity	Q1 mass [Da]	DP [V]	Q3 mass [Da]	CE [V]	CXP [V]	U/S/M
**Plasticizer/plastic components**							
Bisphenol A (BPA)	neg	227.0	-140	133.0<br>116.9	-32<br>-56	-17<br>-21	8.2
Bisphenol AF (BPAF)	neg	335.0	-80	265.1<br>176.8	-32<br>-58	-19<br>-29	12.0
Bisphenol B (BPB)	neg	241.1	-45	212.1<br>210.9	-26<br>-40	-11<br>-15	10.0
Bisphenol C (BPC)	neg	255.0	-70	240.2<br>147.0	-26<br>-34	-29<br>-9	11.8
Bisphenol F (BPF)	neg	198.6	-120	105.0<br>106.0	-28<br>-28	-11<br>-11	6.3
Bisphenol S (BPS)	neg	249.1	-150	108.0<br>155.9	-36<br>-28	-13<br>-13	4.8
Mono-n-butyl phthalate (MBP)	neg	221.0	-5	77.2<br>71.1	-24<br>-20	-5<br>-11	4.0-4.3/3.6-4.1/4.1-4.9
Mono-2-ethylhexyl phthalate (MEHP)	neg	277.0	-40	134.0<br>127.1	-20<br>-20	-9<br>-11	8.4-9/8.2-8.5/8.2- (9.5*)
N-butylbenzenesulfonamide	neg	211.9	-30	140.9<br>64.8	-30<br>-24	-9<br>-29	9.0
Benzyl butyl phthalate	pos	313.0	36	91.0<br>149.0	45<br>19	10<br>12	15.1
Dibutyl phthalate	pos	279.0	11	148.9<br>204.8	21<br>11	16<br>20	15.2
Tetrabromobisphenol A (TBPA)	neg	542.6	-110	445.8<br>419.8<br>417.9	-45<br>-53<br>-56	-28<br>-23<br>-33	15.0
**Perfluorinated alkylated substances**							
Perfluorooctanoic acid (PFOA)	neg	412.9	-20	368.9<br>168.9	-18<br>-22	-19<br>-13	6.8-7.1/6.7-6.9/7-7.7
Perfluorooctanesulfonic acid (PFOS)	neg	498.8	-80	80.0<br>98.9	-126<br>-92	-37<br>-3	11-11.4/10.7-11/11-(12.3*)
**Industrial side products and pesticides**							
2-Naphthol	neg	143.0	-20	115.0<br>114.0	-34<br>-42	-13<br>-17	7.4
Methiocarb	pos	226.0	31	168.7<br>121.1	13<br>31	18<br>16	10.8
Prochloraz	pos	375.9	21	307.8<br>265.7	17<br>23	42<br>16	14.6
2-tert-Butylphenol (2-tert-BP)	neg	149.0	-85	132.9<br>93.0	-32<br>-30	-7<br>-11	13.3
4-Octylphenol (4-OP)	neg	205.1	-15	105.9<br>118.9	-28<br>-44	-19<br>-1	15.5
4-tert-Octylphenol (4-tert-OP)	neg	205.1	-15	134.0<br>133.0	-24<br>-36	-11<br>-19	15.1
Fenarimol	pos	330.9	121	267.9<br>189.1	31<br>59	18<br>12	11.9
Nonylphenol	neg	219.1	-20	133.0<br>134.0	-42<br>-24	-11<br>-9	15.4
**Endogenous estrogens**							
Estrone (E1)	neg	269.4	-140	144.8<br>143.0	-50<br>-50	-2<br>-2	10.5
Estradiol (E2)	neg	271.1	-50	144.9<br>183.0	-52<br>-52	-15<br>-2	8.9/8.6-8.9/8.9
Estradiol-17-glucuronide (E2-17-GlcA)	neg	447.1	-55	113.0<br>271.0	-30<br>-36	-9<br>-21	4.0/3.8-3.9/3.9-4.2
Estradiol-3-sulfate (E2-3-sulfate)	neg	351.1	-170	271.1<br>80.0	-44<br>-62	-17<br>-11	4.7/4.5-4.7/4.7-4.9
Estriol (E3)	neg	287.0	-140	171.0<br>145.0	-48<br>-52	-27<br>-9	5.0
16-Epiestriol (16EpiE3)	neg	287.1	-95	145.0<br>171.1	-50<br>-52	-11<br>-11	6.0
16-α-Hydroxyestrone (16OHE1)	neg	285.1	-160	145.0<br>143.0	-46<br>-73	-11<br>-19	6.1
17-Epiestriol (17EpiE3)	neg	287.1	-125	145.0<br>143.1	-52<br>-64	-13<br>-9	6.3
2-Methoxy estrone (2MeOE1)	neg	299.1	-30	284.2<br>159.9	-32<br>-54	-21<br>-9	11.6
2-Methoxy estradiol(2MeOE2)	neg	301.1	-45	286.1<br>285.2	-34<br>-48	-17<br>-21	10.0
4-Methoxy estrone (4MeOE1)	neg	299.1	-55	284.1<br>283.2	-28<br>-42	-23<br>-19	11.1

Compound	Polarity	Q1 mass [Da]	DP [V]	Q3 mass [Da]	CE [V]	CXP [V]	Retention time [min] U/S/M
		MS/MS parameters					
4-Methoxy estradiol (4MeOE2)	neg	301.1	-40	286.1<br>284.9	-28<br>-46	-17<br>-23	9.3
2-Hydroxy estradiol (2OHE2)	neg	287.0	-155	147.0<br>161.1	-58<br>-54	-15<br>-13	7.0/7.2/7.0
4-Hydroxy estrone (4OHE1)	neg	285.2	-130	161.1<br>159.1	-50<br>-66	-15<br>-9	7.9-8.9
**Phytoestrogens and metabolites**							
8-Prenylnaringenin	neg	339.0	-15	219.0<br>119.0	-26<br>-38	-19<br>-15	11.6-11.7
Coumestrol	neg	267.0	-50	265.9<br>211.0	-38<br>-38	-15<br>-11	6.4
Daidzein	neg	253.0	-10	224.0<br>207.9	-36<br>-40	-17<br>-13	5.2
Enterodiol	neg	301.1	-90	253.1<br>271.1	-32<br>-32	-15<br>-19	5.2
Enterolactone	neg	297.1	-105	253.1<br>107.0	-28<br>-32	-43<br>-11	6.8
Equol	neg	241.1	-45	120.9<br>118.9	-18<br>-28	-13<br>-5	6.5
Formononetin	neg	267.0	-90	252.0<br>223.0	-28<br>-42	-17<br>-17	7.9
Genistein	neg	269.0	-5	133.1<br>132.0	-38<br>-54	-11<br>-21	6.4
Glycitein	pos	285.0	166	270.0<br>241.9	33<br>43	22<br>14	5.4
Isoxanthohumol	pos	355.1	31	179.0<br>298.9	35<br>21	12<br>18	8.4
Matairesinol	neg	269.0	-65	83.0<br>137.1	-26<br>-30	-11<br>-11	6.5
Resveratrol	neg	227.0	-45	185.0<br>142.9	-26<br>-30	-11<br>-17	5.0
Xanthohumol	pos	355.0	76	179.0<br>299.0	29<br>17	10<br>20	14.7
**Mycoestrogens and metabolites**							
Alternariol	neg	257.0	-110	213.0<br>215.0	-32<br>-34	-13<br>-13	6.8
Alternariol monomethyl ether	neg	271.1	-95	256 .0<br>228.0	-32<br>-39	-13<br>-20	11.4
α-Zearalanol (α-ZAL)	neg	321.1	-120	277.1<br>303.1	-30<br>-30	-18<br>-20	8.8-8.9
β-Zearalanol (β-ZAL)	neg	321.1	-120	277.1<br>303.1	-30<br>-30	-18<br>-20	7.5
α-Zearalenol (α-ZEL)	neg	319.1	-80	160.0<br>174.0	-44<br>-37	-13<br>-9	9.2
β-Zearalenol (β-ZEL)	neg	319.1	-115	275.2<br>160.0	-30<br>-44	-15<br>-13	7.7
α-Zearalenol-14-glucuronide (α-ZEL-14-GlcA)	neg	495.0	-20	319.2<br>113.0	-38<br>-26	-23<br>-13	4.1/3.8-3.9/3.9-4.2
β-Zearalenol-14-glucuronide (β-ZEL-14-GlcA)	neg	495.1	-25	319.2<br>112.9	-38<br>-28	-23<br>-55	3.7/3.5-3.6/3.6-3.8
Zearalanone (ZAN)	neg	319.2	-75	275.1<br>161.0	-35<br>-38	-20<br>-15	11.5
Zearalenone (ZEN)	neg	317.1	-75	175.0<br>131.0	-34<br>-40	-9<br>-9	11.7
Zearalenone-14-glucuronide (ZEN-14-GlcA)	neg	493.0	-35	317.0<br>112.9	-36<br>-26	-25<br>-13	4.4/4.1-4.2/4.3-4.5
Zearalenone-14-sulfate (ZEN-14-sulfate)	neg	397.1	-80	316.9<br>175.0	-28<br>-46	-23<br>-15	5.3/5.1-5.3/5.3-5.6
**Personal care product ingredients, pharmaceuticals and metabolites**							
Benzophenone 1	neg	213.0	-60	91.0<br>134.9	-34<br>-28	-11<br>-17	9.0
Benzophenone 2	neg	245.1	-40	135.0<br>109.0	-22<br>-30	-23<br>-15	5.5
Benzylparaben	neg	227.0	-20	92.1<br>136.0	-30<br>-20	-13<br>-13	10.5
Butylparaben	neg	193.1	-25	92.0<br>137.0	-32<br>-22	-7<br>-21	10.1
Ethylparaben	neg	164.9	-30	92.1<br>136.9	-30<br>-20	-9<br>-15	6.1
Isobutylparaben	neg	193.0	-55	92.0<br>135.9	-32<br>-24	-7<br>-11	9.9
Methylparaben	neg	151.0	-35	92.0<br>135.9	-26<br>-20	-13<br>-15	4.9
Propylparaben	neg	179.0	-55	92.0<br>93.0	-30<br>-26	-9<br>-11	7.8
Ethinylestradiol	neg	295.1	-155	145.0<br>159.0	-48<br>-48	-13<br>-17	10.2

Compound	MS/MS parameters						Retention time [min]
	Polarity	Q1 mass [Da]	DP [V]	Q3 mass [Da]	CE [V]	CXP [V]	U/S/M
3-Benzylidencamphor (3-BC)	pos	241.1	76	91.0<br>165.1	51<br>51	10<br>14	15.2
4-methylbenzylidencamphor (4-MBC)	pos	255.1	81	104.8<br>141.2	37<br>59	12<br>12	15.5
Octyl methoxycinnamate (OMC)	pos	291.1	156	179.0<br>161.1	13<br>25	10<br>18	16.1
p-Hydroxybenzoic acid (pOHBA)	neg	136.9	-5	92.9<br>65.0	-18<br>-36	-17<br>-9	0.7-2.4/0.7-1.4/0.9-2.7
Triclosan	neg	286.8	-5	35.0	-38	-15	14.9
**Phytotoxins**							
Anisodamine	pos	306.1	106	140.1<br>122.1	33<br>35	10<br>10	3.6/3.6-3.8/3.2-3.5
Aristolochic acid I	pos	358.9	26	298.0<br>296.0	15<br>15	18<br>8	5.3-5.6/4.7-5.2/5.3-(6.0)*
Aristolactam I	pos	293.9	191	279.0<br>250.9	37<br>47	18<br>22	10.5
Jacobine	pos	352.0	136	155.0<br>119.9	37<br>39	18<br>14	3.5-3.7/3.6-4.4/3.3-3.6
Jacobine-N-oxide	pos	368.0	116	296.0<br>120.1	33<br>43	18<br>8	3.1
Riddelliin	pos	350.0	86	120.0<br>138.0	37<br>39	14<br>16	3.4/3.4-3.8/3.1-3.3
Riddelliin-N-oxide	pos	366.0	126	120.0<br>118.0	41<br>39	14<br>12	3.1
Scopolamine	pos	304.0	41	156.2<br>138.1	23<br>25	10<br>16	3.6-3.7/3.7-4.2/3.4-3.6
**Disinfection by-products**							
Bromoacetic acid	neg	136.9<br>139.0 (isotope)	-10	78.9<br>81.0	-13<br>-13	-26<br>-26	0.8/0.7-0.9/0.7
Dibromoacetic acid	neg	216.8	-5	172.8<br>80.9	-14<br>-32	-27<br>-9	0.9/0.9-1/0.9-1.2
Dichloroacetic acid	neg	126.9	-10	83.0<br>35.0	-32<br>-12	-5<br>-15	0.8/0.8/0.8-1
**Food processing by-products**							
Acrylamide	pos	72.0	26	44.0<br>55.0	49<br>41	14<br>10	0.9-1.0
5-Hydroxymethylfurfural (HMF)	pos	126.9	21	109.0<br>81.0	15<br>23	14<br>10	2.4
5-Hydroxymethyl-2-furanoic acid (HMFA)	neg	140.9	-5	97.1<br>69.0	-10<br>-18	-15<br>-9	0.7/0.7/0.7-1.1
N-Nitrosodimethylamine (NDMA)	pos	74.9	26	43.1<br>58.0<br>44.0	21<br>17<br>17	20<br>8<br>6	1.4
PhIP	pos	225.1	51	210.1<br>140.1	41<br>69	10<br>14	5.3/5.3/5.1-5.3
**Air pollutants**							
Cotinine	pos	176.9	86	80.0<br>98.0	31<br>27	10<br>18	3.1
Trans-3-hydroxy cotinine	pos	193.0	86	80.1<br>134.0	33<br>27	10<br>14	2.7/2.7/2.2-2.7
1-Hydroxy pyrene	neg	217.0	-115	189.0<br>188.0	-46<br>-48	-13<br>-25	14.2
3-Hydroxy phenanthrene	neg	193.0	-135	165.0<br>164.2	-40<br>-46	-13<br>-13	11.4-11.5
**Internal Standards**							
$^{13}C_{12}$ Bisphenol A (BPA)	neg	239.0	-140	139.0	-38	-21	8.2
$^{13}C_{18}$ Zearalenone (ZEN)	neg	335.2	-110	185.1	-34	-13	11.7
$^{13}C_{2}$ mono-2-ethylhexyl phthalate (MEHP)	neg	281.1	-40	136.9	-20	-9	8.4-9/8.2-8.5/8.2- (9.5)*
$^{13}C_{2}$ mono-*n*-butyl phthalate (MBP)	neg	225.0	-5	79.0	-24	-5	4.0-4.3/3.6-4.1/4.1-4.9
$^{13}C_{3}$ Estradiol (E2)	neg	274.1	-50	186.0	-52	-2	8.9
$^{13}C_{6}$ 4-tert-Octylphenol (4-tert-OP)	neg	211.1	-15	139.0	-36	-19	15.1
$^{13}C_{6}$ Butylparaben	neg	199.0	-25	98.0	-32	-7	10.1
$^{13}C_{6}$ Ethylparaben	neg	171.0	-30	97.9	-30	-9	6.1
$^{13}C_{6}$ Methylparaben	neg	157.0	-35	97.9	-26	-13	4.9
$^{13}C_{6}$ p-Hydroxybenzoic acid (pOHBA)	neg	142.9	-5	98.9	-18	-17	0.7-2.4/0.7-1.4/0.9-2.7
$^{13}C_{6}$ Propylparaben	neg	185.0	-55	97.9	-30	-9	7.8
$^{13}C_{8}$ Perfluorooctanesulfonic acid (PFOS)	neg	506.8	-80	79.9	-126	-37	11-11.4/10.7-11/11-(12.3)*
$^{13}C_{8}$ Perfluorooctanoic acid (PFOA)	neg	420.9	-20	375.9	-18	-19	6.8-7.1/6.7-6.9/7-7.7
D_4 Genistein	neg	272.9	-5	136.9	-54	-21	6.4

* A retention time shift outside the programmed MRM window was observed during one validation run.

Supplementary Table 3: Spiking levels and extraction recovery results as obtained during in-house validation.
Fortified concentrations at low level (LL) and high level (HL), extraction recovery (R_E), intermediate precision (RSD_R) and repeatability (RSD_r) of the investigated xenobiotics and endogenous estrogens in three matrices. Parameters that could not be determined are displayed as n.d. For a few compounds, the R_E and RSD_R could not be calculated, while the RSD_r was calculated. This was due to no signal detected in at least the low-level fortified samples during the first two validation runs, but successfully-detected peaks during the third validation sequence which were used to assess the repeatability of the method.

Compound	LL/HL [ng mL^{-1}]	Urine			Serum			Breast milk		
		$R_E \pm RSD_R$ (LL) [%]	$R_E \pm RSD_R$ (HL) [%]	RSD_r LL/HL [%]	$R_E \pm RSD_R$ (LL) [%]	$R_E \pm RSD_R$ (HL) [%]	RSD_r LL/HL[%]	$R_E \pm RSD_R$ (LL) [%]	$R_E \pm RSD_R$ (HL) [%]	RSD_r LL/HL [%]
**Plasticizer/plastic components**										
Bisphenol A (BPA)	0.3/3	116 ± 23	94 ± 9	13/7	104 ± 20	89 ± 21	13/7	82 ± 15	81 ± 20	11/10
Bisphenol AF (BPAF)	0.15/1.5	97 ± 5	88 ± 5	4/4	96 ± 10	94 ± 16	2/5	47 ± 22	52 ± 16	13/8
Bisphenol B (BPB)	0.03/0.3	91 ± 15	91 ± 5	16/9	76 ± 17	89 ± 16	8/11	53 ± 47	60 ± 21	55/11
Bisphenol C (BPC)	0.6/6	95 ± 6	92 ± 5	9/4	82 ± 11	88 ± 12	8/5	50 ± 22	56 ± 19	14/8
Bisphenol F (BPF)	0.15/1.5	100 ± 7	98 ± 5	21/5	73 ± 20	81 ± 6	20/6	72 ± 18	81 ± 9	8/4
Bisphenol S (BPS)	0.006/0.06	87 ± 29	90 ± 11	30/7	109 ± 42	90 ± 17	32/7	95 ± 16	83 ± 10	12/9
Mono-n-butyl phthalate (MBP)	1.5/15	95 ± 13	101 ± 12	11/5	138 ± 26	143 ± 28	6/3	78 ± 17	75 ± 13	16/5
Mono-2-ethylhexyl phthalate (MEHP)*	0.45/4.5	n.d.	99 ± 10	n.d./5	n.d.	n.d.	n.d.	47 ± 31	39 ± 14	n.d.
N-butylbenzenesulfonamide	3/30	96 ± 7	92 ± 4	9/2	87 ± 12	88 ± 8	5/4	71 ± 61	69 ± 15	80/5
Benzyl butyl phthalate	0.75/7.5	80 ± 15	68 ± 10	22/13	78 ± 25	65 ± 13	13/9	n.d.	n.d.	n.d.
Dibutyl phthalate	15/150	36 ± 102	35 ± 56	12/73	95 ± 28	50 ± 14	20/13	n.d.	n.d.	n.d.
Tetrabromobisphenol A (TBPA)	0.3/3	91 ± 20	81 ± 19	18/10	56 ± 49	49 ± 20	31/8	n.d.	n.d.	n.d.
**Perfluorinated alkylated substances**										
Perfluorooctanoic acid (PFOA)	0.045/0.45	119 ± 25	90 ± 9	9/8	84 ± 16	82 ± 15	6/8	75 ± 25	59 ± 19	16/5
Perfluorooctanesulfonic acid (PFOS)*	0.045/0.45	100 ± 47	84 ± 21	4/2	n.d.	56 ± 25	6/5	95 ± 18	66 ± 11	n.d.
**Industrial side products and pesticides**										
2-Naphthol	0.09/0.9	95 ± 19	85 ± 10	7/13	96 ± 18	81 ± 4	18/4	76 ± 71	52 ± 19	32/8
Methiocarb**	0.015/0.15	n.d.	83 ± 5	7/5	n.d.	85 ± 7	3/7	38 ± 25	52 ± 16	27/12
Prochloraz**	0.0015/0.015	n.d.	83 ± 15	11/9	n.d.	85 ± 12	30/8	n.d.	n.d.	n.d.
2-tert-Butylphenol (2-tert-BP)	150/1500	n.d.	n.d.	n.d.	5 ± 73	6 ± 35	15/12	20 ± 56	24 ± 49	44/67
4-Octylphenol (4-OP)	30/300	43 ± 82	n.d.	22/n.d.	106 ± 34	101 ± 32	14/10	n.d.	n.d.	n.d.
4-tert-Octylphenol (4-tert-OP)**	4.5/45	n.d.	6 ± 159	12/73	80 ± 11	75 ± 15	6/5	n.d.	26 ± 18	57/21
Fenarimol	0.009/0.09	88 ± 13	85 ± 6	3/6	95 ± 5	92 ± 10	9/4	n.d.	37 ± 24	n.d./13
Nonylphenol	7.5/75	37 ± 92	n.d.	n.d.	121 ± 58	89 ± 21	24/11	n.d.	n.d.	n.d.
**Endogenous estrogens**										
Estrone (E1)	0.009/0.09	129 ± 14	94 ± 13	37/12	82 ± 20	85 ± 14	20/13	n.d.	44 ± 56	n.d./42
Estradiol (E2)	0.09/0.9	107 ± 23	102 ± 12	9/7	76 ± 28	86 ± 13	21/8	n.d.	50 ± 21	n.d./13
Estradiol-17-glucuronide (E2-17-GlcA)	0.15/1.5	n.d.	n.d.	n.d.	n.d.	n.d.	n.d.	77 ± 28	73 ± 9	31/8
Estradiol-3-sulfate (E2-3-sulfate)**	0.045/0.45	n.d.	91 ± 9	45/10	77 ± 21	90 ± 8	12/5	56 ± 41	51 ± 20	36/16
Estriol (E3)	0.09/0.9	95 ± 21	97 ± 7	31/7	104 ± 23	86 ± 7	20/8	60 ± 33	66 ± 12	16/11
16-Epiestriol (16EpiE3)	0.3/3	91 ± 13	95 ± 4	13/4	93 ± 11	83 ± 8	11/6	57 ± 25	60 ± 18	16/9
16-α-Hydroxyestrone (16OHE1)	0.045/0.45	67 ± 20	88 ± 9	27/8	82 ± 18	81 ± 8	18/6	74 ± 30	70 ± 14	11/6
17-Epiestriol (17EpiE3)	0.3/3	94 ± 19	96 ± 8	16/3	86 ± 9	85 ± 7	8/3	57 ± 24	64 ± 14	24/8
2-Methoxy estrone (2MeOE1)	0.075/0.75	97 ± 12	94 ± 5	17/5	97 ± 8	84 ± 10	11/4	43 ± 53	49 ± 17	51/15
2-Methoxy estradiol(2MeOE2)	0.06/0.6	99 ± 12	96 ± 5	8/4	90 ± 11	86 ± 6	8/2	n.d.	46 ± 18	n.d./14
4-Methoxy estrone (4MeOE1)	0.015/0.15	96 ± 16	93 ± 5	13/3	83 ± 12	88 ± 10	10/3	n.d.	53 ± 20	n.d./8

Compound	LL/HL [ng mL-1]	Urine			Serum			Breast milk		
		R_E ± RSD_R (LL) [%]	R_E ± RSD_R (HL) [%]	RSD_r LL/HL [%]	R_E ± RSD_R (LL) [%]	R_E ± RSD_R (HL) [%]	RSD_r LL/HL[%]	R_E ± RSD_R (LL) [%]	R_E ± RSD_R (HL) [%]	RSD_r LL/HL [%]
4-Methoxy estradiol (4MeOE2)	0.03/0.3	95 ± 17	98 ± 5	11/4	91 ± 17	88 ± 6	6/3	27 ± 54	50 ± 19	69/9
4-Hydroxy estrone (4OHE1)**	0.015/0.15	79 ± 21	82 ± 18	20/6	n.d.	n.d.	n.d.	n.d.	40 ± 24	47/7
**Phytoestrogens and metabolites**										
8-Prenylnaringenin	0.09/0.9	88 ± 11	90 ± 6	12/6	87 ± 7	91 ± 11	2/2	39 ± 22	41 ± 25	14/12
Coumestrol	0.015/0.15	96 ± 11	92 ± 5	10/4	94 ± 20	85 ± 8	14/7	58 ± 24	68 ± 19	16/7
Daidzein**	0.015/0.15	98 ± 21	96 ± 5	23/10	84 ± 28	81 ± 11	13/5	n.d.	93 ± 13	42/12
Enterodiol	0.015/0.15	89 ± 15	104 ± 11	12/8	92 ± 13	82 ± 6	4/6	43 ± 64	42 ± 34	46/27
Enterolactone	0.6/6	101 ± 6	97 ± 5	9/5	90 ± 7	88 ± 7	3/2	77 ± 33	83 ± 10	8/5
Equol	0.06/0.6	97 ± 5	98 ± 3	6/3	97 ± 6	85 ± 3	5/1	60 ± 22	62 ± 22	18/10
Formononetin	0.0075/0.075	100 ± 6	94 ± 5	5/4	94 ± 11	91 ± 10	9/2	58 ± 19	66 ± 19	11/8
Genistein	0.015/0.15	107 ± 18	96 ± 13	10/7	71 ± 22	82 ± 15	21/10	184 ± 73	80 ± 18	110/17
Glycitein**	0.015/0.15	n.d.	89 ± 17	21/4	107 ± 15	85 ± 5	14/6	72 ± 24	79 ± 10	13/10
Isoxanthohumol	0.003/0.03	91 ± 17	89 ± 5	7/4	83 ± 23	92 ± 13	9/4	n.d.	57 ± 20	n.d./17
Matairesinol	0.15/1.5	78 ± 13	96 ± 7	19/4	83 ± 14	84 ± 7	11/3	83 ± 19	80 ± 8	12/5
Resveratrol	4.5/45	93 ± 17	92 ± 17	4/1	72 ± 8	77 ± 13	8/7	10 ± 39	10 ± 61	37/31
Xanthohumol	0.3/3	91 ± 17	89 ± 5	7/4	83 ± 23	92 ± 13	9/4	n.d.	57 ± 20	n.d./17
**Mycoestrogens and metabolites**										
Alternariol	0.3/3	94 ± 8	94 ± 4	5/4	91 ± 11	89 ± 10	4/3	61 ± 20	67 ± 17	10/6
Alternariol monomethyl ether	0.015/0.15	94 ± 13	92 ± 5	8/5	66 ± 24	88 ± 15	15/6	n.d.	51 ± 19	n.d./15.4
α-Zearalanol (α-ZAL)	0.15/1.5	97 ± 4	94 ± 5	4/3	96 ± 12	88 ± 9	4/3	44 ± 20	54 ± 20	15/12
β-Zearalanol (β-ZAL)	0.15/1.5	102 ± 4	96 ± 4	7/3	91 ± 6	86 ± 5	6/2	54 ± 22	63 ± 22	13/9
α-Zearalenol (α-ZEL)**	0.006/0.06	n.d.	89 ± 8	14/7	n.d./7	86 ± 14	n.d./8	n.d.	56 ± 30	n.d./15
β-Zearalenol (β-ZEL)	0.3/3	100 ± 6	95 ± 6	16/3	94 ± 5	87 ± 6	5/2	51 ± 21	59 ± 21	12/10
α-Zearalenol-14-glucuronide (α-ZEL-14-GlcA)	0.045/0.45	n.d.	n.d.	n.d.	n.d.	83 ± 12	n.d./7	61 ± 22	59 ± 23	16/15
β-Zearalenol-14-glucuronide (β-ZEL-14-GlcA)	0.045/0.45	n.d.	n.d.	n.d.	n.d.	n.d.	n.d.	64 ± 23	54 ± 13	21/9
Zearalanone (ZAN)	0.09/0.9	98 ± 9	92 ± 4	10/5	88 ± 17	92 ± 9	8/4	n.d.	50 ± 24	16/10
Zearalenone (ZEN)	0.09/0.9	98 ± 11	94 ± 7	7/6	86 ± 8	87 ± 11	6/8	n.d.	57 ± 17	8/10
Zearalenone-14-glucuronide (ZEN-14-GlcA)	0.15/1.5	n.d.	n.d.	n.d.	n.d.	83 ± 5	n.d./6	68 ± 24	76 ± 13	23/12
Zearalenone-14-sulfate (ZEN-14-sulfate)	0.045/0.45	99 ± 9	97 ± 4	9/5	91 ± 7	89 ± 9	5/3	46 ± 43	46 ± 33	22/12
**Personal care product ingredients, pharmaceuticals and metabolites**										
Benzophenone 1	0.06/0.6	98 ± 5	93 ± 4	3/3	96 ± 15	97 ± 13	25/8	58 ± 16	61 ± 20	12/7
Benzophenone 2	0.045/0.45	97 ± 8	95 ± 7	5/5	89 ± 8	88 ± 20	9/23	75 ± 13	80 ± 6	9/6
Benzylparaben	0.0045/0.045	97 ± 7	92 ± 5	7/5	92 ± 6	90 ± 10	5/3	51 ± 19	57 ± 22	23/8
Butylparaben	0.03/0.3	87 ± 5	90 ± 8	4/6	85 ± 7	86 ± 14	4/1	47 ± 28	52 ± 22	18/5
Ethylparaben	0.03/0.3	93 ± 10	94 ± 10	5/8	81 ± 15	87 ± 13	14/5	103 ± 63	71 ± 17	36/4
Isobutylparaben	0.03/0.3	87 ± 5	86 ± 3	3/3	91 ± 9	89 ± 9	4/2	45 ± 23	57 ± 24	26/10
Methylparaben	0.075/0.75	95 ± 13	98 ± 10	8/8	74 ± 20	89 ± 15	18/4	85 ± 33	68 ± 23	26/4
Propylparaben	0.06/0.6	93 ± 7	93 ± 8	5/5	80 ± 11	86 ± 12	6/2	78 ± 36	68 ± 19	29/3
Ethinylestradiol	0.3/3	97 ± 13	94 ± 6	11/6	93 ± 11	88 ± 12	14/5	n.d.	51 ± 18	n.d./7
3-Benzylidencamphor (3-BC)**	45/450	n.d.	n.d.	n.d.	34 ± 16	26 ± 19	10/13	n.d.	27 ± 31	7/5

4-methylbenzylidencamphor (4-MBC)**	4.5/45	n.d.	18 ± 83	13/77	64 ± 16	45 ± 15	9/13	n.d.	n.d.	n.d./7

**Compound**	**LL/HL [ng mL^{-1}]**	**Urine**			**Serum**			**Breast milk**		
		**R_E ± RSD_R (LL) [%]**	**R_E ± RSD_R (HL) [%]**	**RSD_r LL/HL [%]**	**R_E ± RSD_R (LL) [%]**	**R_E ± RSD_R (HL) [%]**	**RSD_r LL/HL[%]**	**R_E ± RSD_R (LL) [%]**	**R_E ± RSD_R (HL) [%]**	**RSD_r LL/HL [%]**
Octyl methoxycinnamate (OMC)	60/600	n.d.	103 ± 32	n.d./22	n.d.	n.d.	n.d.	n.d.	n.d.	n.d.
p-Hydroxybenzoic acid (pOHBA)	15/150	n.d.	n.d.	n.d.	76 ± 43	88 ± 9	86/5	74 ± 18	78 ± 8	7/3
Triclosan	0.3/3	72 ± 12	59 ± 16	13/25	100 ± 14	75 ± 17	10/6	n.d.	25 ± 39	n.d./8
**Phytotoxins**										
Anisodamine	0.015/0.15	n.d.	87 ± 14	n.d./7	106 ± 23	88 ± 8	10/3	43 ± 26	57 ± 12	97/19
Aristolochic acid I*	0.3/3	78 ± 23	88 ± 16	23/7	73 ± 39	91 ± 18	6/7	n.d.	46 ± 13	n.d.
Aristolactam I	0.15/1.5	93 ± 10	86 ± 6	8/4	84 ± 11	84 ± 15	3/4	n.d.	33 ± 19	n.d./14
Jacobine	0.075/0.75	n.d.	71 ± 24	n.d./5	90 ± 11	89 ± 7	10/3	45 ± 29	42 ± 27	25/12
Jacobine-N-oxide	0.015/0.15	n.d.	82 ± 14	n.d./13	102 ± 24	83 ± 15	13/6	29 ± 72	24 ± 44	53/19
Riddelliin	0.09/0.9	n.d.	76 ± 23	n.d./9	88 ± 13	91 ± 8	10/3	43 ± 25	53 ± 22	11/27
Riddelliin-N-oxide	0.06/0.6	n.d.	76 ± 23	n.d./10	101 ± 13	91 ± 9	10/6	39 ± 44	39 ± 31	29/29
Scopolamine	0.0045/0.045	102 ± 17	90 ± 13	16/6	96 ± 4	92 ± 7	2/1	56 ± 23	65 ± 10	16/14
**Disinfection by-products**										
Bromoacetic acid	6/60	n.d.	n.d.	n.d.	n.d.	53 ± 61	n.d./8	n.d.	n.d.	n.d.
Dibromoacetic acid **	4.5/45	n.d.	83 ± 10	11/8	83 ± 10	82 ± 11	3/3	37 ± 39	44 ± 31	34/30
Dichloroacetic acid **	22.5/225	n.d.	98 ± 7	16/6	101 ± 11	87 ± 11	8/2	29 ± 49	31 ± 42	41/62
**Food processing by-products**										
Acrylamide	30/300	n.d.	86 ± 23	n.d./6	64 ± 13	64 ± 10	6/8	28 ± 33	34 ± 16	26/59
5-Hydroxymethylfurfural (HMF)**	10.5/105	n.d.	27 ± 21	84/26	75 ± 12	74 ± 9	4/5	n.d.	19 ± 87	n.d./54
5-Hydroxymethyl-2-furanoic acid (HMFA)	60/600	n.d.	n.d.	n.d.	86 ± 10	91 ± 5	11/5	50 ± 17	52 ± 14	5/18
N-Nitrosodimethylamine (NDMA)	90/900	n.d.	n.d.	n.d.	n.d.	n.d.	n.d.	n.d.	n.d.	n.d.
PhIP	0.03/0.3	96 ± 13	99 ± 9	8/4	93 ± 4	86 ± 10	3/1	55 ± 22	68 ± 19	11/14
**Air pollutants**										
Cotinine	0.45/4.5	86 ± 16	96 ± 7	13/3	n.d.	n.d.	n.d.	n.d.	n.d.	68/57
Trans-3-hydroxy cotinine**	0.15/1.5	n.d.	91 ± 16	45/5	n.d.	n.d.	74/5	n.d.	n.d.	136/46
1-Hydroxy pyrene	0.6/6	91 ± 25	93 ± 16	15/8	62 ± 20	75 ± 17	12/8	104 ± 48	35 ± 29	27/15
3-Hydroxy phenanthrene	0.045/0.45	92 ± 8	88 ± 6	7/4	87 ± 12	86 ± 15	10/5	n.d.	50 ± 32	n.d./15

* Data from breast milk originate from two evaluated validations only, as a retention time shift outside the programmed MRM window was observed in the last validation run. Therefore, no repeatability could be calculated.
** R_E and RSD_R could not be calculated, while the RSDr was calculated. This was due to no signal being detected in at least one of the low-level fortified samples during the first two validation runs, but successfully-detected peaks during the third validation sequence which was used to assess the repeatability of the method.

Supplementary Table 4: Calibration parameters, limits of quantitation (LOQ), matrix effects and ion ratios as obtained during in-house validation. Average (n=3) regression coefficients, calibrated concentration ranges, limits of quantification (LOQs), signal suppression or enhancement (SSE) and ion ratios (given for the quantitated transition) of the investigated xenobiotics and endogenous estrogens in urine (U), serum (S) and breast milk (M). Parameters that could not be determined are displayed as n.d.

Compound	Regression coefficient R^2	Calibration range [ng mL^{-1}]	LOQ [ng mL^{-1}]			SSE ± s [%]			Ion ratio
	U/S/M	U/S/M	U	S	M	U	S	M	U/S/M
**Plasticizer/plastic components**									
Bisphenol A (BPA)	0.992/0.985/0.991	0.03-10/0.1-10/0.1-10	0.21	0.57	0.4	103 ± 6	58 ± 5	88 ± 7	6/5/5
Bisphenol AF (BPAF)	0.993/0.995/0.989	0.015-5	0.038	0.075	0.041	87 ± 7	48 ± 6	32 ± 1	6/6/6
Bisphenol B (BPB)	0.999/0.993/0.993	0.03-1	0.022	0.043	0.028	100 ± 6	52 ± 3	68 ± 5	49/47/46
Bisphenol C (BPC)	0.999/0.998/0.993	0.06-20/0.2-20/0.2-20	0.22	0.85	0.24	99 ± 6	50 ± 3	51 ± 3	97/99/95
Bisphenol F (BPF)	0.996/0.996/0.996	0.05-5	0.064	0.068	0.028	119 ± 18	80 ± 12	114 ± 16	8/7/8
Bisphenol S (BPS)	0.99/0.994/0.988	0.002-0.2	0.036	0.0086	0.0078	57 ± 15	87 ± 6	96 ± 17	30/28/28
Mono-n-butyl phthalate (MBP)	0.998/0.998/0.987	0.15-50	0.34	0.2	0.16	103 ± 10	96 ± 15	88 ± 3	48/50/51
Mono-2-ethylhexyl phthalate (MEHP)*	0.996/n.d./0.992	0.045-15/n.d./0.045-4.5	0.047	n.d.	3.8	100 ± 8	n.d.	11 ± 4	52/51/51
N-butylbenzenesulfonamide	0.998/0.997/0.992	0.3-100	1.2	1	1.5	100 ± 3	95 ± 3	82 ± 2	94/95/94
Benzyl butyl phthalate	0.98/0.949/n.d.	0.075-25/0.075-25/n.d.	0.18	0.66	n.d.	43 ± 7	11 ± 3	n.d.	75/72/70
Dibutyl phthalate	0.934/0.983/n.d.	1.5-500/1.5-500/n.d.	3.1	7.6	n.d.	56 ± 11	17 ± 7	n.d.	53/53/57
Tetrabromobisphenol A (TBPA) **	0.988/0.986/n.d.	0.03-10/0.3-10/n.d.	0.021	0.33	n.d.	44 ± 4	70 ± 44	n.d.	111/99/106; 97/86/77
**Perfluorinated alkylated substances**									
Perfluorooctanoic acid (PFOA)	0.994/0.992/0.98	0.015-1.5/0.0045-1.5/0.0045-0.45	0.22	0.18	0.092	131 ± 13	73 ± 12	110 ± 14	43/50/41
Perfluorooctanesulfonic acid (PFOS)*	0.993/0.991/0.997	0.045-1.5/0.015-1.5/0.015-1.5	0.049	0.14	0.016	108 ± 10	42 ± 3	86 ± 6	17/17/17
**Industrial side products and pesticides**									
2-Naphthol	0.998/0.994/0.991	0.009-3/0.01-3/0.009-3	0.019	0.029	0.021	130 ± 12	85 ± 9	103 ± 14	2/2/1
Methiocarb	0.992/0.992/0.991	0.05-0.5/0.015-0.5/0.015-0.5	0.017	0.036	0.015	72 ± 3	67 ± 9	35 ± 5	69/70/68
Prochloraz	0.992/0.992/0.955	0.0005-0.05/0.0015-0.05/0.015-0.05	0.0003	0.00076	0.05	58 ± 1	35 ± 5	11 ± 10	13/15/16
2-tert-Butylphenol (2-tert-BP)	0.998/0.998/0.995	15-5000	34	63	98	97 ± 2	60 ± 1	37 ± 6	88/88/87
4-Octylphenol (4-OP)	0.991/0.967/0.928	3-300/3-1000/30-1000	1.4	2.3	24	27 ± 12	42 ± 15	1.3 ± 0.4	1/1/2
4-tert-Octylphenol (4-tert-OP)	0.991/0.993/0.992	0.45-150/0.45-150/15-45	0.14	3.3	40	77 ± 7	18 ± 1	4 ± 1	99/99/97
Fenarimol	0.982/0.997/0.993	0.003-0.3/0.003-3/0.009-0.3	0.0028	0.0055	0.0077	48 ± 12	56 ± 3	13 ± 0.3	30/27/30
Nonylphenol	0.989/0.951/n.d.	0.75-250/7.5-250/n.d.	3.2	1.6	n.d.	33 ± 18	53 ± 14	n.d.	13/13/14
**Endogenous estrogens**									
Estrone (E1)	0.993/0.993/0.989	0.003-0.3/0.009-0.3/0.009-0.3	0.0059	0.0085	0.0069	111 ± 15	69 ± 11	41 ± 2	29/28/26
Estradiol (E2)	0.996/0.995/0.98	0.09-3	0.051	0.076	0.093	104 ± 6	79 ± 7	46 ± 5	85/80/79
Estradiol-17-glucuronide (E2-17-GlcA)	n.d./0.983/0.996	n.d./1.5-5/0.15-5	n.d.	4.9	0.91	n.d.	232 ± 77	198 ± 77	n.d./103/74
Estradiol-3-sulfate (E2-3-sulfate)	0.996/0.994/0.997	0.045-1.5/0.015-1.5/0.0045-1.5	0.2	0.012	0.012	103 ± 18	96 ± 5	91 ± 4	12/4/5
Estriol (E3)	0.995/0.996/0.995	0.09-3/0.03-3/0.09-3	0.12	0.058	0.11	122 ± 23	117 ± 17	110 ± 17	76/75/74
16-Epiestriol (16EpiE3)	0.995/0.995/0.997	0.3-10	0.23	0.13	0.19	129 ± 19	103 ± 27	98 ± 15	92/91/93
16-α-Hydroxyestrone (16OHE1)	0.996/0.995/0.996	0.045-1.5	0.096	0.034	0.037	122 ± 16	104 ± 21	98 ± 13	35/38/39
17-Epiestriol (17EpiE3)	0.998/0.997/0.998	0.03-10/0.1-10/0.1-10	0.12	0.087	0.11	128 ± 19	103 ± 22	92 ± 11	56/57/57
2-Methoxy estrone (2MeOE1)	0.999/0.998/0.989	0.025-2.5/0.075-2.5/0.075-2.5	0.046	0.05	0.045	134 ± 31	94 ± 24	43 ± 2	4/4/4
2-Methoxy estradiol(2MeOE2)	0.998/0.998/0.986	0.006-2/0.02-2/0.06-2	0.018	0.027	0.032	134 ± 36	100 ± 30	42 ± 5	2/2/2
4-Methoxy estrone (4MeOE1)	0.998/0.995/0.988	0.005-0.5/0.015-0.5/0.015-0.5	0.016	0.011	0.009	106 ± 10	75 ± 6	44 ± 3	58/60/59
4-Methoxy estradiol (4MeOE2)	0.999/0.998/0.99	0.01-1/0.03-1/0.03-1	0.024	0.015	0.013	107 ± 13	87 ± 8	44 ± 2	46/44/46
4-Hydroxy estrone (4OHE1)	0.997/n.d./0.991	0.005-0.5/0.15-5/0.015-0.5	0.013	n.d.	0.0073	162 ± 29	126 ± 13	134 ± 33	15/x/16
**Phytoestrogens and metabolites**									

Compound	Regression coefficient R^2	Calibration range [ng mL^{-1}]	LOQ [ng mL^{-1}]			SSE ± s [%]			Ion ratio
	U/S/M	U/S/M	U	S	M	U	S	M	U/S/M
8-Prenylnaringenin	0.99/0.997/0.993	0.009-3/0.009-3/0.03-3	0.01	0.023	0.015	87 ± 7	62 ± 25	23 ± 4	59/58/60
Coumestrol	0.997/0.996/0.996	0.005-0.5/0.015-0.5/0.015-0.5	0.01	0.0061	0.0048	193 ± 30	103 ± 13	137 ± 6	43/41/42
Daidzein	0.99/0.986/0.982	0.005-0.5/0.005-0.5/0.0015-0.5	0.025	0.015	0.0083	142 ± 27	118 ± 18	139 ± 34	101/102/103
Enterodiol	0.993/0.997/0.996	0.015-0.5/0.005-0.5/0.0015-0.5	0.18	0.0045	0.0014	112 ± 5	91 ± 8	105 ± 13	20/27/26
Enterolactone	0.999/0.999/0.998	0.06-20	1.1	0.17	0.17	114 ± 9	84 ± 13	115 ± 12	77/77/78
Equol	0.999/0.998/0.999	0.006-2/0.02-2/0.02-2	0.0076	0.028	0.014	132 ± 16	85 ± 20	114 ± 13	65/68/67
Formononetin	0.9995/0.998/0.998	0.00075-0.25	0.0028	0.001	0.00067	109 ± 9	84 ± 20	76 ± 4	27/27/27
Genistein	0.992/0.989/0.985	0.005-0.5/0.015-0.5/0.005-0.5	0.011	0.019	0.019	154 ± 26	90 ± 17	129 ± 20	40/43/42
Glycitein	0.992/0.994/0.998	0.015-5/0.05-5/0.0015-0.5	0.13	0.057	0.0023	55 ± 24	162 ± 55	162 ± 70	51/54/54
Isoxanthohumol	0.992/0.993/0.996	0.001-0.1/0.003-0.1/0.001-0.1	0.0072	0.011	0.0095	48 ± 8	50 ± 21	55 ± 7	42/41/42
Matairesinol	0.997/0.996/0.999	0.05-5/0.05-5/0.15-5	0.65	0.11	0.13	162 ± 41	145 ± 51	149 ± 29	68/68/68
Resveratrol	0.995/0.991/0.998	1.5-150/0.45-45/0.45-150	2.2	0.92	0.3	201 ± 78	116 ± 85	381 ± 224	70/74/71
Xanthohumol	0.941/0.895/0.98	0.03-10/0.1-10/0.1-3	0.052	0.14	0.22	16 ± 9	34 ± 14	6 ± 3	81/78/82
**Mycoestrogens and metabolites**									
Alternariol	0.999/0.999/0.999	0.1-10	0.16	0.14	0.076	138 ± 19	81 ± 21	102 ± 13	73/72/71
Alternariol monomethyl ether	0.997/0.997/0.983	0.015-0.5	0.0077	0.011	0.019	97 ± 6	57 ± 16	20 ± 1	24/25/23
α-Zearalanol (α-ZAL)	0.9995/0.999/0.993	0.05-5/0.015-5/0.05-5	0.1	0.068	0.092	106 ± 11	88 ± 12	54 ± 4	28/28/28
β-Zearalanol (β-ZAL)	0.999/0.999/0.998	0.015-5/0.05-5/0.05-5	0.13	0.06	0.053	113 ± 11	98 ± 5	84 ± 3	27/28/28
α-Zearalenol (α-ZEL)	0.995/0.993/0.99	0.02-0.2/0.02-0.2/0.06-0.2	0.012	0.0084	0.063	104 ± 6	71 ± 18	41 ± 7	94/99/84
β-Zearalenol (β-ZEL)	0.999/0.999/0.997	0.1-10	0.4	0.24	0.16	114 ± 10	90 ± 7	77 ± 4	45/45/45
α-Zearalenol-14-glucuronide (α-ZEL-14-GlcA)	n.d./0.997/0.997	n.d./0.45-1.5/0.045-1.5	n.d.	0.66	0.02	n.d.	140 ± 23	132 ± 21	n.d./89/90
β-Zearalenol-14-glucuronide (β-ZEL-14-GlcA)	n.d./n.d./0.997	n.d./n.d./0.045-1.5	n.d.	0.42	0.04	n.d.	73 ± 67	169 ± 46	n.d./72/91
Zearalanone (ZAN)	0.999/0.996/0.992	0.09-3	0.08	0.2	0.12	98 ± 6	71 ± 19	37 ± 5	69/68/72
Zearalenone (ZEN)	0.998/0.996/0.989	0.009-3/0.03-3/0.09-3	0.025	0.032	0.086	98 ± 7	53 ± 7	35 ± 6	76/77/76
Zearalenone-14-glucuronide (ZEN-14-GlcA)	n.d./0.996/0.996	n.d./0.5-5/0.15-5	n.d.	1.2	0.093	n.d.	155 ± 12	143 ± 33	n.d./83/83
Zearalenone-14-sulfate (ZEN-14-sulfate)	0.999/0.999/0.998	0.015-1.5/0.015-1.5/0.0045-1.5	0.15	0.021	0.015	156 ± 20	122 ± 11	102 ± 6	14/14/14
**Personal care product ingredients, pharmaceuticals and metabolites**									
Benzophenone 1	0.999/0.997/0.994	0.006-2	0.0054	0.017	0.012	106 ± 3	79 ± 28	65 ± 2	99/98/99
Benzophenone 2	0.999/0.995/0.999	0.015-1.5/0.0045-1.5/0.0045-1.5	0.018	0.027	0.0096	149 ± 48	108 ± 7	152 ± 53	59/61/62
Benzylparaben	0.999/0.998/0.993	0.0045-0.15	0.00055	0.0011	0.00058	101 ± 4	70 ± 20	46 ± 1	75/75/73
Butylparaben	0.999/0.999/0.987	0.003-1/0.01-1/0.003-1	0.0072	0.0096	0.0099	104 ± 1	64 ± 0	60 ± 1	30/30/30
Ethylparaben	0.998/0.996/0.994	0.003-1/0.003-1/0.01-1	0.0039	0.019	0.041	128 ± 4	108 ± 6	110 ± 3	65/66/67
Isobutylparaben	0.999/0.998/0.996	0.01-1/0.01-1/0.003-1	0.0083	0.0073	0.0086	103 ± 3	76 ± 18	63 ± 1	37/36/37
Methylparaben	0.998/0.994/0.992	0.0075-2.5	0.048	0.035	0.028	137 ± 5	121 ± 7	117 ± 7	38/39/40
Propylparaben	0.999/0.997/0.992	0.006-2	0.011	0.0081	0.01	111 ± 2	88 ± 4	92 ± 3	34/34/33
Ethinylestradiol	0.999/0.999/0.993	0.1-10/0.1-10/0.3-10	0.04	0.12	0.21	102 ± 6	65 ± 3	42 ± 3	64/66/64
3-Benzylidencamphor (3-BC)	0.983/0.967/0.985	4.5-450/4.5-1500/4.5-450	0.3	2	6.1	61 ± 6	17 ± 3	4 ± 1	69/73/73
4-methylbenzylidencamphor (4-MBC)	0.987/0.936/0.979	0.45-150/0.45-150/1.5-150	0.43	0.41	14	49 ± 6	37 ± 6	4 ± 4	71/73/71
Octyl methoxycinnamate (OMC)	0.965/0.972/n.d.	200-600/60-200; 200-600; 600-2000/n.d.	160	260	730	9 ± 1	90 ± 79	n.d.	18/28/0
p-Hydroxybenzoic acid (pOHBA)	n.d./0.988/0.995	n.d./5-500/1.5-500	n.d.	9.8	7.6	n.d.	531 ± 109	403 ± 283	4/4/5
Triclosan	0.997/0.97/0.985	0.03-10/0.03-10/0.3-10	0.035	0.048	1.0	64 ± 7	24 ± 6	2 ± 0.3	no qualifier
**Phytotoxins**									
Anisodamine	0.993/0.997/0.996	0.05-0.5/0.005-0.5/0.0015-0.5	0.066	0.008	0.001	35 ± 11	98 ± 6	95 ± 5	15/12/12

Compound	Regression coefficient R^2	Calibration range [ng mL^{-1}]	LOQ [ng mL^{-1}]			SSE ± s [%]			Ion ratio
	U/S/M	U/S/M	U	S	M	U	S	M	U/S/M
Aristolochic acid I*	0.984/0.994/0.993	0.3-10/0.3-10/0.1-10	0.33	0.24	0.44	37 ± 13	75 ± 20	24 ± 7	44/43/45
Aristolactam I	0.997/0.994/0.987	0.015-5/0.05-5/0.15-5	0.016	0.019	0.2	46 ± 8	44 ± 7	3 ± 0.3	41/41/41
Jacobine	0.972/0.995/0.998	0.075-2.5/0.075-2.5/0.025-2.5	0.4	0.042	0.035	28 ± 10	100 ± 8	94 ± 8	102/91/92
Jacobine-N-oxide	0.994/0.99/0.997	0.05-0.5/0.015-0.5/0.0015-0.5	0.061	0.0086	0.002	16 ± 5	75 ± 17	87 ± 2	43/42/40
Riddelliin	0.989/0.996/0.998	0.3-3/0.09-3/0.03-3	1.2	0.18	0.12	30 ± 13	104 ± 13	95 ± 7	74/76/77
Riddelliin-N-oxide	0.995/0.997/0.996	0.6-2/0.06-2/0.006-2	1	0.04	0.021	17 ± 6	84 ± 10	86 ± 4	92/88/87
Scopolamine	0.992/0.998/0.999	0.00045-0.15	0.0025	0.00042	0.00014	32 ± 10	101 ± 7	98 ± 4	85/92/90
**Disinfection by-products**									
Bromoacetic acid	n.d./0.985/n.d.	n.d./2-200/n.d.	n.d.	79	n.d.	n.d.	2 ± 1.6	n.d.	no qualifier
Dibromoacetic acid	0.993/0.998/0.998	4.5-150/0.45-150/0.45-150	32	1.8	2.7	35 ± 6	80 ± 23	93 ± 18	15/18/17
Dichloroacetic acid	0.996/0.996/0.992	22.5-750/2.25-750/7.5-750	60	10	6.6	29 ± 3	32 ± 4	74 ± 9	63/67/57
**Food processing by-products**									
Acrylamide	0.987/0.998/0.995	30-1000/10-1000/10-1000	92	5.3	22	23 ± 3	56 ± 5	73 ± 11	57/60/57
5-Hydroxymethylfurfural (HMF)	0.991/0.992/0.991	3.5-350/1.05-350/1.05-350	29	16	5.6	23 ± 11	91 ± 13	84 ± 17	34/34/33
5-Hydroxymethyl-2-furanoic acid (HMFA)	n.d./0.993/0.994	n.d./60-2000/6-2000	n.d.	150	22	n.d.	2 ± 0.4	57 ± 29	27/36/26
N-Nitrosodimethylamine (NDMA)**	0.996/0.999/0.999	30-3000/90-3000/30-3000	240	230	170	76 ± 4	103 ± 4	105 ± 2	31/33/35; 8/8/9
PhIP	0.991/0.994/0.999	0.003-1/0.01-1/0.003-1	0.011	0.01	0.0042	136 ± 6	140 ± 32	190 ± 60	15/16/15
**Air pollutants**									
Cotinine	0.989/0.765/0.994	0.045-15/0.45-15/0.045-15	0.89	0.054	0.11	20 ± 8	n.d.	96 ± 3	32/32/32
Trans-3-hydroxy cotinine	0.987/0.979/0.993	0.15-5/0.05-5/0.015-5	2.3	0.065	0.025	20 ± 9	66 ± 27	95 ± 14	95/89/90
1-Hydroxy pyrene	0.989/0.996/0.991	0.06-20/0.2-20/0.2-6	0.19	0.22	1.3	128 ± 42	48 ± 22	10 ± 7	12/13/11
3-Hydroxy phenanthrene	0.997/0.997/0.992	0.015-1.5/0.045-1.5/0.045-1.5	0.021	0.032	0.053	111 ± 6	56 ± 7	22 ± 4	2/2/2

*Data from breast milk originate from two evaluated validations only, as a retention time shift outside the programmed MRM window was observed in the last validation run.
** A second qualifier transition was included.

Supplementary Table 5: Validation outcomes for all included chemicals.

Evaluated parameters are extraction recovery (R_E), intermediate precision (RSD_R) and repeatability (RSD_r) at the low- (LL) and high (HL) fortification level. Compounds fulfilling all parameters are marked with (✓), while for the others the criteria that are out of acceptable range are listed. Retention time (RT) is listed as a parameter outside validation limits if insufficient retention was achieved (RT<1.1 min.) and R^2 is listed if linear calibration was not satisfactory (R^2<0.9) or <3 standard levels were above the limit of detection (LOD) in the specific matrix.

Compound	Summary of validation outcomes pointing out critical parameters			Comment
	Urine	Serum	Breast milk	
**Plasticizer/plastic components**				
Bisphenol A (BPA)	✓	✓	✓	
Bisphenol AF (BPAF)	✓	✓	[R_E LL]*	
Bisphenol B (BPB)	✓	✓	[RSD_R LL, RSD_r LL]*	
Bisphenol C (BPC)	✓	✓	✓	
Bisphenol F (BPF)	✓	✓	✓	
Bisphenol S (BPS)	✓	[RSD_R LL]*	✓	
Mono-n-butyl phthalate (MBP)	✓	R_E LL/HL, RSD_R HL	✓	
Mono-2-ethylhexyl phthalate (MEHP)	[R_E LL]*	R^2	(R_E LL/HL)	Retention time shifted out of MRM window in two LL samples in urine; high matrix contamination did not allow for linear regression in serum; retention time shifted out of MRM window in third breast milk validation, therefore only two batches were evaluated
N-butylbenzenesulfonamide	✓	✓	[RSD_R LL, RSD_r LL]*	
Benzyl butyl phthalate	✓	✓	R^2	High matrix contamination prevented linear regression in breast milk
Dibutyl phthalate	R_E LL/HL, RSD_R LL/HL, RSD_r HL	[RSD_R LL]*	R^2	Matrix contamination in urine prevented evaluation of the fortified samples at the LL; high matrix contamination prevented linear regression in breast milk
Tetrabromobisphenol A (TBPA)	✓	R_E HL, RSD_R LL	R^2	Lower sensitivity in breast milk hindered linear regression calibration
**Perfluorinated alkylated substances**				
Perfluorooctanoic acid (PFOA)	✓	✓	✓	
Perfluorooctanesulfonic acid (PFOS)	[RSD_R LL]*	[R_E LL]*	(✓)	Matrix contamination in serum prevented evaluation of the fortified samples at the LL; retention time shifted out of MRM window in third breast milk validation, therefore only two batches were evaluated
**Industrial side products and pesticides**				
2-Naphthol	✓	✓	[RSD_R LL]*	
Methiocarb	[R_E LL]*	[R_E LL]*	[R_E LL]*	Higher noise level during second validation in all matrices hindered the evaluation of the fortified samples at the LL
Prochloraz	[R_E LL]*	[R_E LL]*	R^2	Higher noise level during second validation in all matrices hindered the evaluation of fortified samples at the LL in all matrices and at the HL in breast milk
4-tert-Octylphenol (4-tert-OP)	R_E LL/HL	✓	R^2	

Compound	Summary of validation outcomes pointing out critical parameters			Comment
	Urine	Serum	Breast milk	
Fenarimol	✓	✓	R_E LL/HL	
Nonylphenol	R_E LL/HL	[R_E LL, RSD_R LL]*	R^2	Matrix contamination in urine prevented the evaluation of the fortified samples at the LL and HL; high matrix contamination prevented linear regression in breast milk
**Phytoestrogens and metabolites**				
8-Prenylnaringenin	✓	✓	R_E LL/HL	
Coumestrol	✓	✓	✓	
Daidzein	✓	✓	[R_E LL]*	Matrix contamination in breast milk prevented the evaluation of the fortified samples at the LL
Enterodiol	✓	✓	R_E LL/HL, RSD_r LL	
Enterolactone	✓	✓	[RSD_R LL]*	
Equol	✓	✓	✓	
Formononetin	✓	✓	✓	
Genistein	✓	✓	[R_E LL, RSD_R LL, RSD_r LL]*	
Glycitein	[R_E LL]*	✓	✓	
Isoxanthohumol	✓	✓	[R_E LL]*	
Matairesinol	✓	✓	✓	
Resveratrol	✓	✓	R_E LL/HL, RSD_R LL, RSD_r LL	
Xanthohumol	✓	✓	[R_E LL]*	
**Mycoestrogens and metabolites**				
Alternariol	✓	✓	✓	
Alternariol monomethyl ether	✓	✓	[R_E LL]*	Matrix contamination in breast milk prevented the evaluation of the fortified samples at the LL
α-Zearalanol (α-ZAL)	✓	✓	[R_E LL]*	
β-Zearalanol (β-ZAL)	✓	✓	✓	
α-Zearalenol (α-ZEL)	[R_E LL]*	✓	R_E LL, RSD_r HL	
β-Zearalenol (β-ZEL)	✓	✓	✓	
α-Zearalenol-14-glucuronide (α-ZEL-14-GlcA)	R^2	[R_E LL]*	✓	High noise level in urine prevented linear regression and the detection of fortified samples
β-Zearalenol-14-glucuronide (β-ZEL-14-GlcA)	R^2	R^2	✓	High noise level in urine and serum prevented linear regression and the detection of fortified samples
Zearalanone (ZAN)	✓	✓	[R_E LL]*	
Zearalenone (ZEN)	✓	✓	[R_E LL]*	
Zearalenone-14-glucuronide (ZEN-14-GlcA)	R^2	[R_E LL]*	✓	High noise level in urine prevented linear regression and the detection of fortified samples
Zearalenone-14-sulfate (ZEN-14-sulfate)	✓	✓	R_E LL/HL, RSD_R LL/HL	
**Personal care product ingredients, pharmaceuticals and metabolites**				

Compound	Summary of validation outcomes pointing out critical parameters			Comment
	**Urine**	**Serum**	**Breast milk**	
Benzophenone 1	✓	✓	✓	
Benzophenone 2	✓	✓	✓	
Benzylparaben	✓	✓	✓	
Butylparaben	✓	✓	[R_E LL]*	
Ethylparaben	✓	✓	[RSD_R LL, RSD_r LL]*	
Isobutylparaben	✓	✓	[R_E LL]*	
Methylparaben	✓	✓	[RSD_R LL]*	
Propylparaben	✓	✓	[RSD_R LL]*	
Ethinylestradiol	✓	✓	[R_E LL]*	
4-methylbenzylidencamphor (4-MBC)	R_E LL/HL	[R_E HL]*	R_E LL/HL	Matrix contamination in urine and breast milk prevented evaluation of the fortified samples at the LL and HL
Triclosan	Selectivity	Selectivity	R_E LL/HL, RSD_R HL, selectivity	As before documented in the literature, only one mass transition could be established
**Phytotoxins**				
Anisodamine	[R_E LL]*	✓	[R_E LL, RSDr LL]*	
Aristolochic acid I	✓	[RSD_R LL]*	(R_E LL/HL)	Retention time shifted out of MRM window in third breast milk validation, therefore only two batches were evaluated
Aristolactam I	✓	✓	[R_E LL]*	
Jacobine	[R_E LL]*	✓	R_E LL/HL	
Jacobine-N-oxide	[R_E LL]*	✓	R_E LL/HL, RSD_R LL/HL, RSD_r LL	
Riddelliin	[R_E LL]*	✓	[R_E LL]*	
Riddelliin-N-oxide	[R_E LL]*	✓	R_E LL/HL, RSD_R LL	
Scopolamine	✓	✓	✓	
**Disinfection by-products**				
Dibromoacetic acid	R_E LL, RT	RT $^+$	R_E LL/HL, RSD_R LL/HL, RSD_r LL/HL RT	
Dichloroacetic acid	R_E LL, RT	RT $^+$	R_E LL/HL, RSD_R LL/HL, RSD_r LL/HL, RT	
**Food processing by-products**				
Acrylamide	R_E LL, RT	✓	R_E LL/HL, RSD_R LL, RSD_r HL, RT	
5-Hydroxymethylfurfural (HMF)	R_E LL/HL, RSD_r HL	✓	R_E LL/HL, RSD_R HL, RSD_r HL,	
5-Hydroxymethyl-2-furanoic acid (HMFA)	R^2	RT $^+$	RT $^+$	High matrix contamination prevented linear regression in urine
PhIP	✓	✓	✓	
**Air pollutants**				
Cotinine	✓	R^2	R_E LL/HL	Matrix contamination in breast milk prevented evaluation of the fortified samples at the LL and HL; high matrix contamination prevented linear regression in serum
Trans-3-hydroxy cotinine	[R_E LL]*	R_E LL/HL	R_E LL/HL	Matrix contamination prevented evaluation of the fortified samples at the LL in urine and at the LL and the HL in serum and breast milk
1-Hydroxy pyrene	✓	✓	R_E HL, RSD_R LL	

Compound	Summary of validation outcomes pointing out critical parameters			Comment
	Urine	Serum	Breast milk	
3-Hydroxy phenanthrene	✓	✓	R_E LL/HL	
**Not validated in any matrix**				
2-tert-Butylphenol (2-tert-BP)	R_E LL/HL	R_E LL/HL, RSD_R LL/HL	R_E LL/HL, RSD_R LL/HL, RSD_r LL/HL,	
4-Octylphenol (4-OP)	R_E LL/HL	RSD_R LL/HL	R^2	
3-Benzylidencamphor (3-BC)	R_E LL/HL	R_E LL/HL	R_E LL/HL	Matrix contamination in breast milk prevented evaluation of the fortified samples at the LL and HL
Octyl methoxycinnamate (OMC)	R^2	R^2	R^2	No linear regression using the same three concentration levels between validations possible in any matrix
p-Hydroxybenzoic acid (pOHBA)	R^2	R_E LL, RSD_r LL, RSD_r LL, RT	RT ++	High matrix contamination prevented linear regression in urine
Bromoacetic acid	R^2	R_E LL, RSD_R HL, RT	R^2	Early elution did not allow selective peak annotation in urine and breast milk
N-Nitrosodimethylamine (NDMA)	R_E LL/HL	R_E LL/HL	R_E LL/HL	
**Endogenous estrogens**				
Estrone (E1)	[R_E LL, RSD_r LL]*	✓	R_E LL/HL, RSD_R HL, RSD_r HL	
Estradiol (E2)	✓	✓	[R_E LL]*	
Estradiol-17-glucuronide (E2-17-GlcA)	R^2	R^2	✓	High noise level in urine and serum prevented linear regression and the detection of fortified samples
Estradiol-3-sulfate (E2-3-sulfate)	[R_E LL]*	✓	[RSD_R LL, RSD_r LL]*	
Estriol (E3)	✓	✓	[RSD_R LL]*	
16-Epiestriol (16EpiE3)	✓	✓	✓	
16-α-Hydroxyestrone (16OHE1)	✓	✓	✓	
17-Epiestriol (17EpiE3)	✓	✓	✓	
2-Methoxy estrone (2MeOE1)	✓	✓	R_E LL/HL, RSD_R LL, RSD_r LL	
2-Methoxy estradiol(2MeOE2)	✓	✓	R_E LL/HL	
4-Methoxy estrone (4MeOE1)	✓	✓	[R_E LL]*	
4-Methoxy estradiol (4MeOE2)	✓	✓	[R_E LL, RSD_R LL, RSD_r LL]*	
4-Hydroxy estrone (4OHE1)	✓	R^2	R_E LL/HL	Sensitivity in serum too low for chosen calibration range and quantification of fortified samples

[+] Disinfection by-products and HMFA were not effectively retained on the column; nonetheless peak annotation was successful and selective at the calibrated ranges. Thus, the quantitative validation was considered successful.

[++]While quantitative validation was successful for pOHBA, the decision was made to exclude the compound as partially successfully validated in breast milk because retention times were too variable between batches and individual samples resulting in difficult peak annotation for unknown samples without employing standard addition.

*All validation requirements were fulfilled at one fortification level.

* All validation requirements were fulfilled at one fortification level.

Supplementary Table 6: Comparison of sensitivities with published multi-class methods.

Compound	Matrix	LOQ [ng mL^{-1}]	LOQ [ng mL^{-1}](published method)	Published method	Comment
Bisphenol A (BPA)	Urine*	0.21	0.33*	Heffernan et al, *Talanta* 151, 224-233, (**2016**)[2]	19 compounds total, 2 chemical classes
Bisphenol AF (BPAF)		0.038	0.017*		
Bisphenol B (BPB)		0.022	0.87*		
Bisphenol F (BPF)		0.064	1.3*		
Bisphenol S (BPS)		0.036	0.22*		
Mono-n-butyl phthalate (MBP)		0.34	0.17*		
Mono-2-ethylhexyl phthalate (MEHP)		0.047	0.067*		
Bisphenol A (BPA)	Urine	0.21	0.1	Rocha et al, *Talanta* 183, 94-101, (**2018**)[3]	21 compounds total, 3 chemical classes
Bisphenol AF (BPAF)		0.038	0.04		
Bisphenol F (BPF)		0.064	0.25		
Bisphenol S (BPS)		0.036	0.07		
Benzophenone 1		0.0054	0.1		
Benzophenone 2		0.018	0.07		
Benzylparaben		0.00055	0.1		
Butylparaben		0.0072	0.1		
Ethylparaben		0.0039	0.05		
Methylparaben		0.048	0.05		
Propylparaben		0.011	0.1		
Triclosan		0.035	0.5		
Bisphenol A (BPA)	Serum/ Plasma+	0.57	0.042	Kolatorova et al, *Talanta* 174, 21-28, (**2017**)[4]	12 compounds total, 3 chemical classes, application of derivatization
Bisphenol AF (BPAF)		0.075	0.15		
Bisphenol F (BPF)		0.068	0.044		
Bisphenol S (BPS)		0.0086	0.055		
Benzylparaben		0.0011	0.20		
Butylparaben		0.0096	0.13		
Ethylparaben		0.019	0.15		
Methylparaben		0.035	0.17		
Propylparaben		0.0081	0.17		
Estrone (E1)		0.0085	0.11		
Estradiol (E2)		0.076	0.006		
Estriol (E3)		0.058	0.009		
Bisphenol A (BPA)	Urine	0.21	0.2	Bocato et al, *Environ Res* 189 (**2020**)[5]	21compounds total, 4 chemical classes
Bisphenol AF (BPAF)		0.038	0.2		
Bisphenol F (BPF)		0.064	0.15		
Bisphenol S (BPS)		0.036	0.14		
Benzophenone 1		0.0054	0.05		
Benzophenone 2		0.018	0.1		
Benzylparaben		0.00055	0.08		
Butylparaben		0.0072	0.03		
Ethylparaben		0.0039	0.1		
Methylparaben		0.048	0.15		
Propylparaben		0.011	0.02		
Triclosan		0.035	0.5		
Bisphenol A (BPA)	Urine	0.21	0.2	Chen et al, *Chromatographia* 1415-1421 (**2019**)[6]	13 compounds total, 4 chemical classes
Benzylparaben		0.00055	0.05		
Butylparaben		0.0072	0.04		
Ethylparaben		0.0039	0.04		
Methylparaben		0.048	0.04		
Propylparaben		0.011	0.04		
Triclosan		0.035	0.12		

+ As no published multi-class assay for serum was found in literature, the sensitivity of our assay in serum could only be compared to the sensitivity of the assay by Kolatorova et al. which was developed for plasma.

Supplementary References

1 Preindl, K. *et al.* A Generic Liquid Chromatography-Tandem Mass Spectrometry Exposome Method for the Determination of Xenoestrogens in Biological Matrices. *Anal Chem* **91**, 11334-11342, doi:10.1021/acs.analchem.9b02446 (2019).

2 Heffernan, A. L. *et al.* Rapid, automated online SPE-LC-QTRAP-MS/MS method for the simultaneous analysis of 14 phthalate metabolites and 5 bisphenol analogues in human urine. *Talanta* **151**, 224-233, doi:https://doi.org/10.1016/j.talanta.2016.01.037 (2016).

3 Rocha, B. A., de Oliveira, A. R. M. & Barbosa, F., Jr. A fast and simple air-assisted liquid-liquid microextraction procedure for the simultaneous determination of bisphenols, parabens, benzophenones, triclosan, and triclocarban in human urine by liquid chromatography-tandem mass spectrometry. *Talanta* **183**, 94-101doi:10.1016/j.talanta.2018.02.052 (2018).

4 Kolatorova Sosvorova, L. *et al.* Determination of selected bisphenols, parabens and estrogens in human plasma using LC-MS/MS. *Talanta* **174**, 21-28, doi:10.1016/j.talanta.2017.05.070 (2017).

5 Bocato, M. Z. *et al.* A fast-multiclass method for the determination of 21 endocrine disruptors in human urine by using vortex-assisted dispersive liquid-liquid microextraction (VADLLME) and LC-MS/MS. *Environmental Research* **189**, 109883, doi:https://doi.org/10.1016/j.envres.2020.109883 (2020).

6 Chen, X. *et al.* Development and Validation of HPLC–MS/MS Method for the Simultaneous Determination of 8-Hydroxy-2′-deoxyguanosine and Twelve Cosmetic Phenols in Human Urine. *Chromatographia* **82**, 1415-1421, doi:10.1007/s10337-019-03757-2 (2019).
