# Supplementary material for: Next-generation biomonitoring of the early-life chemical exposome in neonatal and infant development
Source: Nat Commun. 2022 May 12;13:2653. doi: 10.1038/s41467-022-30204-y (PMC9098442; doi:10.1038/s41467-022-30204-y)
Supplement: Supplementary file 3 — Description of Additional Supplementary Information [file 41467_2022_30204_MOESM3_ESM.pdf]

Description of Additional Supplementary Files

File Name: Supplementary Data 1
Description: Results of neonate plasma analysis and population characteristics.

File Name: Supplementary Data 2
Description: Comparison of three neaonate plasma sample before and after treatment with β-glucuronidase and sulfatase (Helix pomatia).

File Name: Supplementary Data 3
Description: Results of breast milk analysis.

File Name: Supplementary Data 4
Description: Results of breast milk analysis after enzymatic hydrolysis by β-glucuronidase and sulfatase (Helix pomatia).

File Name: Supplementary Data 5
Description: Comparison of analyte concentarations in breast milk samples before and after treatment with β-glucuronidase and sulfatase (Helix pomatia).

File Name: Supplementary Data 6
Description:. Comparison of analyte recoveries after spiking different initial plasma volumes.

File Name: Supplementary Data 7
Description: Spearman correlation of chemicals detected in breast milk.
