# Supplementary material for: Next-generation biomonitoring of the early-life chemical exposome in neonatal and infant development
Source: Nat Commun. 2022 May 12;13:2653. doi: 10.1038/s41467-022-30204-y (PMC9098442; doi:10.1038/s41467-022-30204-y)
Supplement: Supplementary file 11 — Reporting Summary [file 41467_2022_30204_MOESM11_ESM.pdf]

nature portfolio

Corresponding author(s): Benedikt Warth

Last updated by author(s): Mar 19, 2022

Reporting Summary

Nature Portfolio wishes to improve the reproducibility of the work that we publish. This form provides structure for consistency and transparency in reporting. For further information on Nature Portfolio policies, see our Editorial Policies and the Editorial Policy Checklist.

Statistics

For all statistical analyses, confirm that the following items are present in the figure legend, table legend, main text, or Methods section.

n/a	Confirmed	
☐	☒	The exact sample size (*n*) for each experimental group/condition, given as a discrete number and unit of measurement
☐	☒	A statement on whether measurements were taken from distinct samples or whether the same sample was measured repeatedly
☒	☐	The statistical test(s) used AND whether they are one- or two-sided<br>*Only common tests should be described solely by name; describe more complex techniques in the Methods section.*
☒	☐	A description of all covariates tested
☐	☒	A description of any assumptions or corrections, such as tests of normality and adjustment for multiple comparisons
☐	☒	A full description of the statistical parameters including central tendency (e.g. means) or other basic estimates (e.g. regression coefficient) AND variation (e.g. standard deviation) or associated estimates of uncertainty (e.g. confidence intervals)
☒	☐	For null hypothesis testing, the test statistic (e.g. *F*, *t*, *r*) with confidence intervals, effect sizes, degrees of freedom and *P* value noted<br>*Give P values as exact values whenever suitable.*
☒	☐	For Bayesian analysis, information on the choice of priors and Markov chain Monte Carlo settings
☒	☐	For hierarchical and complex designs, identification of the appropriate level for tests and full reporting of outcomes
☐	☒	Estimates of effect sizes (e.g. Cohen's *d*, Pearson's *r*), indicating how they were calculated

Our web collection on statistics for biologists contains articles on many of the points above.

Software and code

Policy information about availability of computer code

Data collection	Data collection was conducted using LC-MS based technology (LC: 1290 Infinity II, Agilent; MS: QTrap 6500+, Sciex) and processed using Sciex OS (version 1.6, Sciex)
Data analysis	Additional statistical analysis and calculations were done in Excel (version 2010, Microsoft). Plots were generated using GraphPad PRISM (version 8.0.1; Graphpad Software, Inc.) and figures were constructed in Inkscape (version 1.0.1; Inkscape). Chemical structures were drawn using ChemDraw (version 12.0.2; PerkinElmer).

For manuscripts utilizing custom algorithms or software that are central to the research but not yet described in published literature, software must be made available to editors and reviewers. We strongly encourage code deposition in a community repository (e.g. GitHub). See the Nature Portfolio guidelines for submitting code & software for further information.

Data

Policy information about availability of data

All manuscripts must include a data availability statement. This statement should provide the following information, where applicable:

- Accession codes, unique identifiers, or web links for publicly available datasets
- A description of any restrictions on data availability
- For clinical datasets or third party data, please ensure that the statement adheres to our policy

The authors declare that the findings of this study are available within the article and the Supplementary Information and Source Data files. Raw data of the LC-MS measurements of the two sample cohorts are accessible via the MetaboLights repository (MTBLS2442).

Field-specific reporting

Please select the one below that is the best fit for your research. If you are not sure, read the appropriate sections before making your selection.

- [x] Life sciences
- [ ] Behavioural & social sciences
- [ ] Ecological, evolutionary & environmental sciences

For a reference copy of the document with all sections, see nature.com/documents/nr-reporting-summary-flat.pdf

Life sciences study design

All studies must disclose on these points even when the disclosure is negative.

Sample size	No sample size calculations were needed. The sample sizes was sufficient for the application of the method in first proof-of concept experiments with no pre-determined standards concerning minimum number of samples. Rational for sample choice: The neonate cohort was selected as a prime example of a susceptible subgroup as early life chemical exposure may have adverse downstream effects in further development. Breast milk was chosen as it functions as a direct vehicle for potential chemical contamination (at very low concentrations) to the infant and is therefore also relevant for investigations regarding early-life development.
Data exclusions	No data was excluded.
Replication	The method was thoroughly validated according to common guidelines (European Commission Decision Nr. 657/2002). Both sample cohorts (infant plasma, breast milk) were measured once, but, as described, the experimental findings were confirmed for certain analytes using additional enhanced product ion scans.
Randomization	No experimental groups were allocated in this work. The run order of the longitudinal breast milk samples was randomized.
Blinding	No information (sex, name, exact age/weight, clinical conditions) on the premature infant cohort (n=21) was given to the authors who conducted chemical analysis. The breast milk samples (n=86) were randomized by allocating random numbers between (101-186) prior to sample handover. After analysis, the random numbers were tracked back to the respective days of sampling (note: as noted in the SI, for 2 two breast milk samples the date of sampling could unfortunately not be tracked back).

Reporting for specific materials, systems and methods

We require information from authors about some types of materials, experimental systems and methods used in many studies. Here, indicate whether each material, system or method listed is relevant to your study. If you are not sure if a list item applies to your research, read the appropriate section before selecting a response.

Materials & experimental systems

n/a	Involved in the study	
x		Antibodies
x		Eukaryotic cell lines
x		Palaeontology and archaeology
x		Animals and other organisms
	x	Human research participants
x		Clinical data
x		Dual use research of concern

Methods

n/a	Involved in the study	
x		ChIP-seq
x		Flow cytometry
x		MRI-based neuroimaging

Human research participants

Policy information about studies involving human research participants

Population characteristics	The study population consists of 21 extremely premature infants (ten female & eleven male infants; gestational age 25.6 ± 1.2 weeks; birth weight 780 ± 149 gram) which were delivered via C-section (n=13) or vaginally (n=8) at the Medical University of Vienna. All infants survived and were discharged at home with a corrected gestational age of 39 ± 3.4 weeks and discharge weight of 2860 ± 657 gram. Breast milk samples were collected from a 31-year old Austrian mother (weight: 54 kg) who consumed her regular diet throughout the experimental time frame. She reported a high tea consumption to support milk production.
Recruitment	Both sample sets (neonate cohort, longitudinal breast milk) have been used in other studies before as described in the Methods section (Seki et al. 2021 and Braun et al. 2020). This had no impact on the study design or results. Informed consent was obtained from all human research participants.
Ethics oversight	The recruitment of the infant cohort was approved by the ethics committee of the Medical University of Vienna (no. 1348/2017), approval of the parents was given. The breast milk and urine sampling was approved by the ethics committee of the University of Vienna (no. 00157).

Note that full information on the approval of the study protocol must also be provided in the manuscript.
